# Supplementary material for: Quality of life and its related psychological problems during coronavirus pandemic
Source: PLoS One. 2022 Oct 31;17(10):e0276841. doi: 10.1371/journal.pone.0276841 (PMC9621443; doi:10.1371/journal.pone.0276841)
Supplement: Attachment — Submitted filename: PONE-D-21-30356_R1.pdf [file pone.0276841.s004.pdf]

PLOS ONE

Quality of life and its related psychological problems during coronavirus pandemic

--Manuscript Draft--

**Manuscript Number:**	PONE-D-21-30356R1
**Article Type:**	Research Article
**Full Title:**	Quality of life and its related psychological problems during coronavirus pandemic
**Short Title:**	Psychological problems during coronavirus
**Corresponding Author:**	Zahra Hosseini<br>Hormozgan University of Medical Sciences<br>BandarAbbas, IRAN, ISLAMIC REPUBLIC OF
**Keywords:**	Contamination Obsessions, Coronavirus Pandemic, Health Anxiety, Perceived Stress, Quality of Life, Washing Compulsions
**Abstract:**	Background: The prevalence of coronavirus disease 2019 (COVID-19) has endangered the psychological health of individuals. This study aimed to assess the quality of life and its related psychological problems during coronavirus pandemic.<br>Methods: In this cross-sectional study, 559 citizens above the age of 16 years, in Isfahan and Bandar Abbas cities in Iran were selected with a convenient sampling method. An online questionnaire was used to collect the data, which consisted of five sections: demographic information, short health anxiety inventory (SHAI), perceived stress scale (PSS), world health organization quality of life questionnaire (WHOQOL-BREF) and Padua inventory. Data were analyzed using statistical tests including t-test, path analysis and structural equation modeling (SEM) using SPSS 24 and Amos 21 statistical software.<br>Results: There were significant positive relationships between health anxiety and perceived stress (r=0/513), obsessive-compulsive disorder and perceived stress (r=0/19), obsessive-compulsive disorder and health anxiety (r=0/439). Also there were significant negative relationships between obsessive-compulsive disorder and quality of life (r=-0/222), health anxiety and quality of life (r=-0/524), perceived stress and quality of life (r=-0/941).<br>Conclusions: According to our findings, health anxiety, perceived stress and obsessive-compulsive disorder are among the most common psychological problems during coronavirus pandemic which affects the quality of life. Therefore, prevention and treatment of these psychological consequences is one of the most important priorities in the global crisis of Covid-19 disease.
**Order of Authors:**	Atefeh Homayuni
	Zahra Hosseini
	Nahid Shahabi
	Roghayeh Ezati Rad
	Farah Moayyedi
**Response to Reviewers:**	Dear Editor in Chief,<br>Journal of PLOS ONE<br>Thank you for giving us this opportunity to revise the manuscript entitled: "Quality of life and its related psychological problems during coronavirus pandemic". The corrections have been made in the manuscript based on Comments.<br><br>We used the yellow color to highlight the requested revisions by the honorable reviewer 1, the bright green to highlight the requested revisions by the honorable reviewer 2, the dark yellow color to highlight the requested revisions by the honorable reviewer 3, and the turquoise color to highlight the common requested revisions by all of the honorable reviewers. We also used the pink color to highlight the requested revisions (journal requirements).<br><br>I hope they would be satisfactory.

Powered by Editorial Manager® and ProduXion Manager® from Aries Systems Corporation

I wish all the best for you
Shirin Hosseini, Ph.D
Corresponding Author

Reviewer Comments:
Honorable reviewer 1:
1.Please highlight your contribution and novelty of this manuscript with accuracy in the introduction part before the arrangement description.
•Our thanks to the honorable reviewer for your attention. Some explanations about the importance of this study and its novelty were presented in the last paragraph in the Introduction section.
•Regarding the importance and novelty of this study and the necessity of doing it, the following can be mentioned:
1.Coronavirus disease 2019 is a new subject and its effects are diverse in different people. This study has examined various psychological variables and their relationships with quality of life during coronavirus pandemic (especially health anxiety and its relationship with obsessive-compulsive disorder) which has been less studied in other studies. This study also has examined stress and health anxiety separately and few studies have addressed these two types of emotional problems separately.
2.This study concerns a population very affected by the Covid-19 pandemic. Vaccination against covid-19 started later in Iran than in other countries in the world. Delay in starting vaccination, people's negligence in observing health protocols, their resistance to vaccination and other reasons, led to higher prevalence and mortality due to the coronavirus in Iran, and as a result, imposed extra psychological pressure on people.
3.This research was conducted in two provinces of Hormozgan and Isfahan. These cities were more vulnerable to coronavirus, for some reasons: Isfahan is an industrial city and a city with a lot of tourist attractions and Hormozgan is a city with commercial ports. The hot weather, the impossibility of proper ventilation especially during the summer, and social gathering in closed environments led to the prevalence of covid-19 in Hormozgan. Besides, high humidity made it difficult for people to use face masks outdoors. In addition, in Iran, the cultural background of people, who are known to hospitable, was also influential in the increased prevalence of this disease. Generally, hospitality is more prominent in southern provinces of Iran, especially in Hormozgan province. All of this contributes to the high prevalence of covid-19 and further psychological problems in these cities.

2. The literature and theoretical background should improve and add more relevant studies e.g. (latest) to grab and display more contemporary literature critically.
•Our thanks to the honorable reviewer for your attention. According to the honorable reviewer's suggestion, the literature and theoretical background was improved and more relevant studies were added.

3. Please check your manuscript for errors. On first glance at your manuscript, we noticed several grammar errors. Please check the whole paper carefully or have it professionally edited.
•Our thanks to the honorable reviewer for your attention. According to the honorable reviewer's suggestion, the manuscript was professionally edited.

4. In the discussion part, you must contact the results, and some of the content needs to be rewritten.
•Our thanks to the honorable reviewer for your attention. Some changes made in the discussion section. We added sub-headings to the Discussion section to guide the readers through this section. We wrote the discussion section as follows: we summarized the important findings in the first paragraph. We presented the findings separately in several paragraphs and compared them with the results of previous studies. And finally we explained the obtained findings and explained why such a result was achieved.

5. It is suggested to use advanced statistical methods to add the influencing factors on the psychological changes of the population. For example, the potential influencing factors were analyzed by logistic regression.
•Our thanks to the honorable reviewer for your attention. We used path analysis and

structural equation modeling (SEM) to test the relationships between the variables. Regarding the suggestion of honorable reviewer (using logistic regression), it should be noted that logistic regression is used to predict a multi-category dependent variable. However, "quality of life" as a dependent variable in this study, is not a multi-category variable. Based on the explanations provided, does the honorable reviewer suggest a specific statistical test?

6. Note the order of Table 3 and table 4 in the article.
•Our thanks to the honorable reviewer for your attention. The order of table 3 and table 4 in the article was modified.

Honorable Reviewer 2:
1.This study entitled "Quality of life and its related psychological problems during coronavirus pandemic" aimed to assess that relationship in a population of adult people in Iran. This topic has been extensively covered in the literature over the past two years. The correlation between quality of life and mental health has been extensively studied to date. Although this study concerns a population very affected by the covid-19 pandemic, the sample studied does not seem to fully correspond to the population from which it is extracted.
•Our thanks to the honorable reviewer for your attention. Regarding the importance and novelty of this study and the necessity of doing it, the following can be mentioned:
4.Coronavirus disease 2019 is a new subject and its effects are diverse in different people. This study has examined various psychological variables and their relationships with quality of life during coronavirus pandemic (especially health anxiety and its relationship with obsessive-compulsive disorder) which has been less studied in other studies. This study also has examined stress and health anxiety separately and few studies have addressed these two types of emotional problems separately.
5.As the honorable reviewer pointed out, this study concerns a population very affected by the Covid-19 pandemic. Vaccination against covid-19 started later in Iran than in other countries in the world. Delay in starting vaccination, people's negligence in observing health protocols, their resistance to vaccination, economic problems caused by the coronavirus and other reasons, led to higher prevalence and mortality due to the coronavirus in Iran, and as a result, imposed extra psychological pressure on people.
6.This research was conducted in two provinces of Hormozgan and Isfahan. These cities were more vulnerable to coronavirus, for some reasons: Isfahan is an industrial city and a city with a lot of tourist attractions and Hormozgan is a city with commercial ports. The hot weather, the impossibility of proper ventilation especially during the summer, and social gathering in closed environments led to the prevalence of covid-19 in Hormozgan. Besides, high humidity made it difficult for people to use face masks outdoors. In addition, in Iran, the cultural background of people, who are known to hospitable, was also influential in the increased prevalence of this disease. Generally, hospitality is more prominent in southern provinces of Iran, especially in Hormozgan province. All of this contributes to the high prevalence of covid-19 and further psychological problems in these cities.
Some explanations about the importance of this study and its novelty were presented in the last paragraph in the Introduction section.

2. In addition, the demographic characteristics collected are not sufficient to give a complete view of the sample.
•Our thanks to the honorable reviewer for your attention. The most important demographic characteristics related to the purpose of our study (and based on similar studies) were collected. Does the honorable reviewer have a special demographic characteristic in mind?

3.A big lack of data concerns the period in which the study was conducted, or rather in which the responses from the participants were collected.
•Our thanks to the honorable reviewer for your attention. The data collection was conducted from October 22, 2020 to February 3, 2021.

4.In addition, the convenience sample and the method of recruiting participants represent possible selection biases (the recruitment method has not been clearly specified).

Powered by Editorial Manager® and ProduXion Manager® from Aries Systems Corporation

•Our thanks to the honorable reviewer for your attention. Further details on data collection are provided in Materials and Methods (Sample size and sampling procedure section). It should be noted that due to the prevalence of coronavirus and it severity during the study (data collection) in Iran and the need to compliance with quarantine, we had to collect data through social media. Otherwise, both the researchers and the participants would have to pay heavy fines. In study limitations, we have mentioned that one of the limitations of this study is that the questionnaire survey was conducted only via the internet, which was only available to participants who were literate and had access to the internet.

5.Language must be deeply revised.
•Our thanks to the honorable reviewer for your attention. According to the honorable reviewer's suggestion, language was deeply revised.

6.The Discussion section lack of "linearity"; it is unclear, with statements followed by some bibliographical references, without a logic and concise semantic structure.
•Our thanks to the honorable reviewer for your attention. Some changes made in the discussion section. We added sub-headings to the Discussion section to guide the readers through this section. We wrote the discussion section as follows: we summarized the important findings in the first paragraph. We presented the findings separately in several paragraphs and compared them with the results of previous studies. And finally we explained the obtained findings and explained why such a result was achieved.

7. The statistical results are also presented in an unclear way.
•Our thanks to the honorable reviewer for your attention. Some explanations about the results presented in each table, are provided at the bottom of the table.

8. The declared limitations are quite slight, just as the few strengths of the study were not highlighted (for example the use of validated questionnaires).
•Our thanks to the honorable reviewer for your attention. We highlighted the study strengths and modified the study limitations.

9. The conclusion, finally, lack of strength and "interest".
•Our thanks to the honorable reviewer for your attention. We made some changes in the conclusion section.

Honorable Reviewer 3:
Thank you for giving me the opportunity to review this article. Here are some questions to the authors. I think this manuscript has an appropriate introduction and well justified.

1.There are some repetitive data and statements in the abstract (result) that is better to modify.
Our thanks to the honorable reviewer for your attention. According to the honorable reviewer's suggestion, the repetitive data and statements in the Abstract (result section) was modified.

2. Participants: How authors are certain the sample population have no OCOD? I know it is mentioned in limitations but self-disclosure is not enough.
•Our thanks to the honorable reviewer for your attention. It should be noted that individuals who had no history of taking any psychiatric medication were included in the study. In other words, the authors examined this criterion (not suffering from psychological disorders such as obsessive-compulsive disorder) by asking this question: "Do you have a history of taking psychiatric medications?".

3. I suggest that The importance of this study and its novelty should be explain more.
•Our thanks to the honorable reviewer for your attention. Some explanations about the importance of this study and its novelty were presented in the last paragraph in the Introduction section.
•Regarding the importance and novelty of this study and the necessity of doing it, the following can be mentioned:

Powered by Editorial Manager® and ProduXion Manager® from Aries Systems Corporation

	1.Coronavirus disease 2019 is a new subject and its effects are diverse in different people. This study has examined various psychological variables and their relationships with quality of life during coronavirus pandemic (especially health anxiety and its relationship with obsessive-compulsive disorder) which has been less studied in other studies. This study also has examined stress and health anxiety separately and few studies have addressed these two types of emotional problems separately.<br>2.This study concerns a population very affected by the Covid-19 pandemic. Vaccination against covid-19 started later in Iran than in other countries in the world. Delay in starting vaccination, people's negligence in observing health protocols, their resistance to vaccination and other reasons, led to higher prevalence and mortality due to the coronavirus in Iran, and as a result, imposed extra psychological pressure on people.<br>3.This research was conducted in two provinces of Hormozgan and Isfahan. These cities were more vulnerable to coronavirus, for some reasons: Isfahan is an industrial city and a city with a lot of tourist attractions and Hormozgan is a city with commercial ports. The hot weather, the impossibility of proper ventilation especially during the summer, and social gathering in closed environments led to the prevalence of covid-19 in Hormozgan. Besides, high humidity made it difficult for people to use face masks outdoors. In addition, in Iran, the cultural background of people, who are known to hospitable, was also influential in the increased prevalence of this disease. Generally, hospitality is more prominent in southern provinces of Iran, especially in Hormozgan province. All of this contributes to the high prevalence of covid-19 and further psychological problems in these cities.<br><br>4. In my opinion statistical analyses are very complete and all well detailed.<br>•Our thanks to the honorable reviewer for your attention.<br><br>5. In the discussion perhaps it is missing to indicate future lines of research.<br>•Our thanks to the honorable reviewer for your attention. Some suggestions for future studies were presented in the discussion section, at the end of "study limitations" part.
**Additional Information:**	
**Question**	**Response**
**Financial Disclosure**<br><br>Enter a financial disclosure statement that describes the sources of funding for the work included in this submission. Review the submission guidelines for detailed requirements. View published research articles from *PLOS ONE* for specific examples.<br><br>This statement is required for submission and **will appear in the published article** if the submission is accepted. Please make sure it is accurate.	The authors received no specific funding for this work.

**Unfunded studies**<br>Enter: *The author(s) received no specific funding for this work.*<br><br>**Funded studies**<br>Enter a statement with the following details:<br>• Initials of the authors who received each award<br>• Grant numbers awarded to each author<br>• The full name of each funder<br>• URL of each funder website<br>• Did the sponsors or funders play any role in the study design, data collection and analysis, decision to publish, or preparation of the manuscript?<br>• **NO** - Include this sentence at the end of your statement: *The funders had no role in study design, data collection and analysis, decision to publish, or preparation of the manuscript.*<br>• **YES** - Specify the role(s) played.<br><br>* typeset	
**Competing Interests**<br><br>Use the instructions below to enter a competing interest statement for this submission. On behalf of all authors, disclose any competing interests that could be perceived to bias this work—acknowledging all financial support and any other relevant financial or non-financial competing interests.<br><br>This statement is required for submission and **will appear in the published article** if the submission is accepted. Please make sure it is accurate and that any funding sources listed in your Funding Information later in the submission form are also declared in your Financial Disclosure statement.<br><br>View published research articles from *PLOS ONE* for specific examples.	The authors have declared that no competing interests exist.

**NO authors have competing interests**<br><br>Enter: *The authors have declared that no competing interests exist.*<br><br>**Authors with competing interests**<br><br>Enter competing interest details beginning with this statement:<br><br>*I have read the journal's policy and the authors of this manuscript have the following competing interests: [insert competing interests here]*<br><br>* typeset	
**Ethics Statement**<br><br>Enter an ethics statement for this submission. This statement is required if the study involved:<br><br>• Human participants<br>• Human specimens or tissue<br>• Vertebrate animals or cephalopods<br>• Vertebrate embryos or tissues<br>• Field research<br><br>Write "N/A" if the submission does not require an ethics statement.<br><br>General guidance is provided below. Consult the submission guidelines for detailed instructions. **Make sure that all information entered here is included in the Methods section of the manuscript.**	The present research was approved by the ethics committee of Hormozgan University of medical sciences (#IR.HUMS.REC.1399.439). The participants were informed that participation in the study was voluntary. They were asked to provide a letter of informed consent.

Powered by Editorial Manager® and ProduXion Manager® from Aries Systems Corporation

**Format for specific study types**<br><br>**Human Subject Research (involving human participants and/or tissue)**<br>• Give the name of the institutional review board or ethics committee that approved the study<br>• Include the approval number and/or a statement indicating approval of this research<br>• Indicate the form of consent obtained (written/oral) or the reason that consent was not obtained (e.g. the data were analyzed anonymously)<br><br>**Animal Research (involving vertebrate animals, embryos or tissues)**<br>• Provide the name of the Institutional Animal Care and Use Committee (IACUC) or other relevant ethics board that reviewed the study protocol, and indicate whether they approved this research or granted a formal waiver of ethical approval<br>• Include an approval number if one was obtained<br>• If the study involved *non-human primates*, add *additional details* about animal welfare and steps taken to ameliorate suffering<br>• If anesthesia, euthanasia, or any kind of animal sacrifice is part of the study, include briefly which substances and/or methods were applied<br><br>**Field Research**<br><br>Include the following details if this study involves the collection of plant, animal, or other materials from a natural setting:<br>• Field permit number<br>• Name of the institution or relevant body that granted permission	
**Data Availability**<br><br>Authors are required to make all data underlying the findings described fully available, without restriction, and from the time of publication. PLOS allows rare exceptions to address legal and ethical concerns. See the PLOS Data Policy and FAQ for detailed information.	Yes - all data are fully available without restriction

A Data Availability Statement describing where the data can be found is required at submission. Your answers to this question constitute the Data Availability Statement and **will be published in the article**, if accepted.<br><br>**Important:** Stating 'data available on request from the author' is not sufficient. If your data are only available upon request, select 'No' for the first question and explain your exceptional situation in the text box.<br><br>Do the authors confirm that all data underlying the findings described in their manuscript are fully available without restriction?	
**Describe where the data may be found in full sentences. If you are copying our sample text, replace any instances of XXX with the appropriate details.**<br><br>• If the data are **held or will be held in a public repository**, include URLs, accession numbers or DOIs. If this information will only be available after acceptance, indicate this by ticking the box below. For example: *All XXX files are available from the XXX database (accession number(s) XXX, XXX.*).<br>• If the data are all contained **within the manuscript and/or Supporting Information files**, enter the following: *All relevant data are within the manuscript and its Supporting Information files.*<br>• If neither of these applies but you are able to provide **details of access elsewhere**, with or without limitations, please do so. For example:<br><br>*Data cannot be shared publicly because of [XXX]. Data are available from the XXX Institutional Data Access / Ethics Committee (contact via XXX) for researchers who meet the criteria for access to confidential data.*<br><br>*The data underlying the results presented in the study are available from (include the name of the third party*	All relevant data are within the paper and its Supporting Information files.

and contact information or URL).<br>• This text is appropriate if the data are owned by a third party and authors do not have permission to share the data.<br><br>* typeset	
Additional data availability information:	

Manuscript Click here to access/download;Manuscript;Manuscript .docx

Quality of life and its related psychological problems during coronavirus pandemic

Psychological problems during coronavirus

Atefeh Homayuni[1], Zahra Hosseini[2]*, Nahid Shahabi[3], Roghayeh Ezati Rad[4], Farah Moayyedi[5]

1.Student Research Committee, Hormozgan University of Medical Sciences, Bandar Abbas, Iran.
2. Tobacco and Health Research Center, Hormozgan University of Medical Sciences, Bandar Abbas, Iran.
3. Student Research Committee, Hormozgan University of Medical Sciences, Bandar Abbas, Iran.
4. Student Research Committee, Hormozgan University of Medical Sciences, Bandar Abbas, Iran.
5. Tobacco and Health Research Center, Hormozgan University of Medical Sciences, Bandar Abbas, Iran.

***Corresponding Author**

Email: hosseinishirin@ymail.com (ZH)

Abstract

Background: The prevalence of coronavirus disease 2019 (COVID-19) has endangered the psychological health of individuals. This study aimed to assess the quality of life and its related psychological problems during coronavirus pandemic.

Methods: In this cross-sectional study, 559 citizens above the age of 16 years, in Isfahan and Bandar Abbas cities in Iran were selected with a convenient sampling method. An online questionnaire was used to collect the data, which consisted of five sections: demographic information, short health anxiety inventory (SHAI), perceived stress scale (PSS), world health organization quality of life questionnaire (WHOQOL-BREF) and Padua inventory. Data were analyzed using statistical tests including t-test, path analysis and structural equation modeling (SEM) using SPSS 24 and Amos 21 statistical software.

Results: There were significant positive relationships between health anxiety and perceived stress (r=0/513), obsessive-compulsive disorder and perceived stress (r=0/19), obsessive-compulsive disorder and health anxiety (r=0/439). Also there were significant negative relationships between obsessive-compulsive disorder and quality of life (r=-0/222), health anxiety and quality of life (r=-0/524), perceived stress and quality of life (r=-0/941).

Conclusions: According to our findings, health anxiety, perceived stress and obsessive-compulsive disorder are among the most common psychological problems during coronavirus pandemic which affects the quality of life. Therefore, prevention and treatment of these psychological consequences is one of the most important priorities in the global crisis of Covid-19 disease.

Keywords: Contamination Obsessions, Coronavirus Pandemic, Health Anxiety, Perceived Stress, Quality of Life, Washing Compulsions

Introduction

Coronavirus disease (Covid-19) pandemic was first seen in December 2019 in china. The virus spread rapidly around the world and it was recognized as a pandemic by the World Health Organization in March 2020 [1]. Most symptoms of the Covid-19 include fever, cough, shortness of breath, and gastrointestinal symptoms in some cases. In more severe cases, it may cause Acute Respiratory Distress Syndrome (ARDS) and hypoxic respiratory failure, which is a major cause of death in Covid-19 patients [2].

The first case of this disease in Iran was diagnosed in February 19, 2020, when two cases of Covid-19 were reported in Qom, and then the disease spread throughout the Iran [3]. Based on the World Health Organization, up to September 13, 2021, 5237799 cases were confirmed and a total of 112935 deaths have been confirmed in Iran [4].

Covid-19 has imposed a serious threat to physical health and life and has caused a wide range of psychological and physical problems. Some factors such as fear of being infected with the virus, fear of death, spreading false news and rumors, interference in daily activities, travel prohibitions or restrictions, reduced social relationships (colleagues, friends and family), job and financial problems, and several factors related to these conditions endangered the mental health of people [5]. Thus, ~~the issue of~~ psychological effects of this viral disease on the mental health ~~of people~~ at different levels of society is crucial. ~~All studies, examined~~ the psychological disorders of people in quarantine, have reported ~~many signs of mental damage~~ such as emotional disorder, depression, stress, irritability, insomnia, decreased attention, post-traumatic stress disorder, anger and emotional blunting [6-8].

The Covid-19 pandemic has had great disruptive impacts on communities throughout the world due to fear and stress~~. These fears include~~ fears for unemployment due to Covid 19-related restrictions, financial worries, and health-related worries. These stresses and worries

may reduce the quality of life [9]. The Covid-19 pandemic has also been associated with impaired health-related quality of life among local residents in China [10].

The most common triggers of psychological disorders and stress in people quarantined due to Covid-19 disease have been reported to be fear of being infected or infecting others, long period of quarantine, inadequate support and lack of access to care adequate medicine and food, and ~~ultimately~~ fatigue and boredom due to quarantine and isolation [11]. Zarabadipour et al. showed that access to social response systems, considerable weight change and reluctance to perform daily activities were among the factors, which had a significant relationship with stress scores ~~in~~ ~~the~~ medical staff during Covid-19 pandemic. ~~In this study~~, gender, the presence of high risk elderly person in the family, having a fatal case due to the Covid-19 in the family, exacerbation of the chronic disease, job closure, etc. had significant relationships with stress score [12]. Qi et al. [13] in their study among Chinese adults during the Covid-19 pandemic reported that perceived stress scores were negatively correlated with physical component summary score (r=-0.4) and mental component summary score for health related quality of life (r=-0.6). It indicates that participants who reported higher health-related quality of life, experienced lower levels of perceived stress.

Health anxiety occurs when mild physical changes are interpreted as a sign of a disease (usually a serious and dangerous disease) [14]. Most people experience some degree of health anxiety. This awareness can be a protective factor against health threatening factors and play a major role in identifying the early symptoms of the health issue and enhancing health-promoting behaviors [15]. However, its excessive level can be dangerous and harmful to the person [14]. Recent studies in coronavirus pandemics have shown that the frequency and severity of health anxiety have increased significantly [16]. In the case of infectious diseases or pandemic conditions, people with high health anxiety are prone to misunderstanding and they consider all these changes as evidence for an infectious disease [17]. People with high health anxiety

also engage in a variety of non-adaptive safety behaviors. During this disease outbreak, it may be followed by over-washing of hands, social isolation, and purchasing with fear. Low levels of health anxiety can also have negative effects on health behavior. People who find themselves at low risk for disease are also less likely to change their social behavior and ignore recommendations for social distance. Thus, health anxiety is one of several psychological factors that affect one's way of reaction to the spread of the coronavirus [18]. The results of a cross-sectional study conducted by Abdelghani et al. [19] on health care workers in hospitals of Egypt showed that health anxiety to Covid-19 virus was inversely correlated with all domains of quality of life (physical health, r=-0.409; psychological health, r=-0.453; social relationships, r= -0.224 and environmental health, r=-0.385).

Disease-related obsessive-compulsive disorder is another common psychological disorder during the epidemic. Information provided by various sources about the methods of infection with the virus may increase people fear of this disease infection. Fear is a defense mechanism that a person shows against dangerous situations and includes the basic reactions necessary to survive from these threatening situations. However, fear that is not appropriate to the current situation may lead to a variety of psychological disorders such as obsessive-compulsive disorder, anxiety, depression and panic [20]. According to the American Psychological Association, fear of contamination is the most common obsession. Fear of contamination is often associated with mandatory rituals such as hand washing, cleaning, and taking appropriate action to reduce exposure to contaminated sources [21]. Since observing personal hygiene (regular hand washing, use of masks and gloves) and maintaining social distance are among the most important ways to prevent the spread of Covid-19 virus, the risk of practical obsessions during the Coronavirus epidemic are considered very likely [22]. 60.3% of participants in the Abba-Aji et al.'s study [23] reported onset of obsessive-compulsive disorder symptoms (obsessions related to contamination with dirt, germs or viruses) and 53.8% had

compulsions to wash hands repeatedly or in a special way, during the Covid-19 pandemic. Respondents who showed obsessive-compulsive disorder symptoms only since the start of Covid-19 pandemic were significantly more likely to have moderate/high stress.

Delay in starting vaccination, people's negligence in observing health protocols and their resistance to vaccination~~,~~ led to higher prevalence and mortality due to the coronavirus in Iran, and as a result, imposed extra psychological pressure on people and decreased their quality of life. Residents of Hormozgan (a city with commercial ports) and Isahan (an industrial city and a city with a lot of ~~tourist~~ attractions) provinces are more vulnerable to coronavirus disease. Since, the first step in creating effective interventions to promote people's quality of life during coronavirus is to identify the relevant and predictive psychological factors~~,~~ therefore, the present study was conducted to assess quality of life and its related psychological problems during coronavirus pandemic among people over 16 years old in Bandar Abbas and Isfahan.

Materials and methods

Study design and population

This descriptive-correlational study was conducted from October 22, 2020 to February 3, 2021. The statistical population of this study consisted of residents over 16 years old in Isfahan and Bandar Abbas (in Iran).

Sample size and sampling procedure

The sample size was calculated based on the following formula:

$$n = \left[(z_{1-\alpha/2} + z_{1-\beta})/C\right]^2 + 3 \ , \ C = 0.5 * \ln[(1+r)/(1-r)]$$

Assuming a 5% error, 90% test power and a correlation coefficient of -0.18 in previous studies [24], the sample size was estimated at 320 people based on the formula. Considering the design effect of 1.2 for cluster sampling, the final sample size was estimated at 384.

The inclusion criteria for the sample selection included: literacy, access to ~~the~~ Internet to answer questions, not suffering from psychological disorders such as obsessive-compulsive disorder, the minimum age of 16 years, living in Isfahan or Bandar Abbas and willingness to participate in the study. Exclusion criteria ~~also included~~ people with psychological disorders such as obsessive-compulsive disorder and unwillingness to participate in the study.

Regarding to the existing limitations due to the outbreak of Covid-19 and the impossibility of distributing questionnaires in paper form, online questionnaires were designed to collect the data. Questionnaires were sent to Hormozgan and Isfahan provinces through various social media (WhatsApp, Telegram, Linkedin), email, channels and news agencies, public relations of University of Medical Sciences, Red Crescent, Municipality and University Student Research Committee. The questionnaire link was submitted to the participants via various online channels. After publishing the questionnaire link, the people who received it were asked to complete the questionnaire (if they wished) and send it to other people they know. ~~Finally, the participants registered their answers by clicking the submit button. To emphasize on the greater participation of individuals in the study, messages and links to participate in the study were resent as a reminder two weeks after the first submission. Also, with the cooperation of health centers in the studied provinces, a questionnaire link was sent to all people covered by healthcare centers in villages and cities.~~ On the first page of the questionnaire, the purpose of the study was clearly explained to the ~~study~~ participants and they were reminded that mentioning their first and last names would not be necessary and their information would be kept confidential ~~by the research team~~.

Ethics statement

Ethical approval was received for this study from the Ethics Committee of the Hormozgan University of Medical Sciences (IR.HUMS.REC.1399.439). Written informed consent was

obtained from individuals who participated in this study. For participants between 16-18 years of old, the research ethics committee waived the requirement for parental consent.

Research tools

The following demographic questionnaire and self-administered tools were used to measure variables and collect data. The assessed demographic characteristics were gender, marital status, education level, age ~~and~~ employment status, history of infection with Covid-19, underlying diseases.

1- Short Health Anxiety Inventory (SHAI): This inventory is an 18-item self-report scale. Each of the 18 items consists of four statements, in which individuals select the one that best reflects their feelings during the last six months. Items assess worry about health, awareness of bodily sensations or changes, and feared consequences of having an illness. The responses are scored on a 4-point Likert scale (0-3). This questionnaire enables estimating the magnitude of health anxiety in two components: the probability of getting the disease (14 questions) and the negative consequences of getting the disease (4 questions). Overall scores ranged from 0 to 54, whereby the higher scores indicated the higher health anxiety [25]. Several studies have shown that this scale has good internal consistency, as its internal consistency coefficients ranged from 0.71 to 0.95. In the same studies, the reliability coefficient through test-retest method with a one-week interval has been obtained ~~at~~ 0.90 and it has been shown that this scale can distinguish hypochondriasis patients from non-clinical control groups [26]. In the present study, Cronbach's alpha coefficient for this questionnaire was ~~0~~.855.

Perceived Stress Scale (PSS): The Perceived Stress Scale is a 14-item self-report tool developed by Cohen et al. (1983) to measure general perceived stress, thoughts and feelings about stressful events, control, overcoming, coping with stress, and experienced stress over the past month. On this scale, individuals are asked to indicate their feelings over the past 4 weeks

on a 5-point Likert scale from 0 (never) to 4 (always). For example, "In the past month, how often have you felt that you were unable to control the important things in your life?" This scale measures two subscales: a) Perceived helplessness, which includes items 1, 2, 3, 8, 11, 12, and 14. B) Perceived self-efficacy, which includes items 4, 5, 6, 7, 9, 10, and 13, and these items are scored inversely. The lowest score is zero and the highest score is 56. The higher scores indicated the higher perceived stress. In a study conducted by Cohen et al. (1983), the internal consistency coefficients for each of the subscales and the general PSS were between 0.84 to 0.86 [27]. The reliability coefficients of the internal consistency for this scale were obtained 0.84 to 0.86 through Cronbach's alpha coefficient in two groups of students and one group of smokers in the smoking cessation program. Perceived stress scale is significantly correlated with life events, depressive and physical symptoms, social anxiety, and low life satisfaction [27]. In the present study, Cronbach's alpha coefficient for this questionnaire was 0.887.

World Health Organization Quality of Life Questionnaire (WHOQOL-BREF): The Persian version of the WHOQOL-BREF was used to collect information. This questionnaire is a 26-item instrument consisting of four domains: physical health (7 items), psychological health (6 items), social relationships (3 items) and environmental health (8 items). The first two questions assess the general health and quality of life. Each question is scored on a 5-point Likert scale (1: strongly disagree to 5: strongly agree). The score of each aspect is calculated separately from the total score of its questions, so that score 4 indicates the worst and score 20 indicates the best situation in that aspect [28]. In this study, the standardized version of this questionnaire (developed by Yousefy et al. [29]) was used. Cronbach's alpha coefficient for physical health, psychological health, social relationships and environmental health were 0.81, 0.72, 0.78, and 0.76, respectively. In the present study, Cronbach's alpha coefficient for this questionnaire was 0.932.

Washing and Contamination Obsessions Questionnaire: To measure washing compulsions and contamination obsessions, Padua Questionnaire (Modified by Washington State University) (Obsessive-Compulsive Disorder Scale) was used [30]. Subjects' responses to each item are scored on a 5-point Likert scale (0=not at all, 1=low, 2=somewhat, 3=high, and 4=very high). High scores indicated a high level of obsessive-compulsive disorder in the subject. Van Open [31] obtained obsessive-compulsive disorder coefficients at 0.94 for Padua questionnaire and above 0.80 for its subscales. In the present study, Cronbach's alpha coefficient for this questionnaire was 0.875.

Data Analysis

Data were analyzed in SPSS-24 and Amos-21 statistical software. Demographic characteristics were described using descriptive statistics including frequency and percentage. Mean, standard deviation, minimum and maximum values were calculated for continuous variables. To test the hypotheses and discover the relationships between the variables, t-test, path analysis and structural equation modeling (SEM) were used. The level of significance was considered to be 95% ($p < 0.05$).

Results

A total number of 559 questionnaires were completed and returned. The participants' demographic information is as follows: 21.5% of the participants were male and 78.5% were female. More than half of the participants were married (71.6%) and the rest were single (26.8%), divorced (1.1%) or widow (0.5%). The majority of them had bachelor's degree (42.9%). In relation to job situation, 40.9% of the participants were employees. Only 14.5% of the participants were members of the medical, health and treatment staff and 83.4% of them had no history of chronic diseases (Table 1).

Table 1. Participants' demographic information (N = 559)

Characteristics	Categories	n(%)
Gender	Male	120 (21.5)
	Female	439 (78.5)
Marital status	Single	150 (26.8)
	Married	400 (71.6)
	Widow	3 (0.5)
	Divorced	6 (1.1)
Level of education	High school	36 (6.5)
	Diploma	100 (17.9)
	Associate degree	43 (7.7)
	Bachelor's degree	240 (42.9)
	Master's degree and higher	140 (25)
Job situation	Student	20 (3.6)
	University student	39 (7)
	Unemployed	25 (4.5)
	Housewife	181 (32.4)
	Employee	229 (40.9)
	Self-employed	65 (11.6)
Smoking	Only cigarette	23 (4.1)
	Only hookah	22 (3.9)
	Both of them	5 (0.9)
	None of them	509 (91.1)
Medical staff	Yes	81 (14.5)
	No	478 (85.5)
Chronic diseases	Yes	93 (16.6)
	No	466 (83.4)

Descriptive statistics (mean, standard deviation, minimum and maximum values) of research variables are reported in Table 2.

Table 2. Descriptive statistics of research variables

	Minimum	Maximum	Mean	Std. Deviation
age	16	69	37.34	11.19
Quality of life	37.00	128.00	87.03	15.81
Physical health	8.00	35.00	24.35	4.80
Psychological health	7.00	30.00	19.15	4.56
Social relationships	3.00	15.00	10.01	2.53
Environmental health	11.00	40.00	26.26	5.21
Obsessive-compulsive disorder	.00	40.00	19.70	9.12
Contamination obsessions	.00	24.00	10.69	5.54
Washing compulsions	.00	16.00	9.01	4.53
Health anxiety	.00	47.00	14.15	7.67
Risk of disease	.00	40.00	12.32	6.42
Negative effect of disease	.00	12.00	1.83	2.06
Perceived stress	2.00	54.00	27.4955	8.83518
Perceived helplessness	.00	28.00	13.92	5.41

Perceived self-efficacy	.00	28.00	14.43	4.65

As the results showed, the mean and standard deviation scores of perceived stress were (27.49±8.83), health anxiety (14.15±7.67), obsessive-compulsive disorder (19.7±9.12) and quality of life (87.03±15.81).

The correlation matrix of the variables is presented in Table 3.

Table 3. correlation coefficients of study variables

Variable	1	2	3	4	5	6	7	8	9	10	11	12	13
Perceived stress	1	-0.857**	0.897**	0.338**	0.298**	0.332**	-0.715**	-0.569**	-0.751**	-0.579**	-0.546**	0.127**	0.166**
Perceived self-efficacy	-0.857**	1	-0.541**	-0.231**	-0.186**	-0.279**	0.665**	0.508**	0.67**	0.538**	0.556**	-0.071	-0.094*
Perceived helplessness	0.897**	-0.541**	1	0.355**	0.327**	0.303**	-0.597**	-0.493**	-0.65**	-0.483**	-0.413**	0.147**	0.19**
Health anxiety	0.338**	-0.231**	0.355**	1	0.973**	0.693**	-0.366**	-0.357**	-0.337**	-0.245**	-0.28**	0.334**	0.283**
Risk of disease	0.298**	-0.186**	0.327**	0.973**	1	0.508**	-0.302**	-0.316**	-0.283**	-0.186**	-0.216**	0.328**	0.281**
Negative effect of disease	0.322**	-0.279**	0.303**	0.693**	0.508**	1	-0.424**	-0.346**	-0.375**	-0.335**	-0.371**	0.221**	0.18**
Quality of life	-0.715**	0.665**	-0.597**	-0.366**	-0.302**	-0.424**	1	0.851**	0.902**	0.79**	0.861**	-0.187**	-0.193**
Physical health	-0.569**	0.508**	-0.493**	-0.357**	-0.316**	-0.346**	0.851**	1	0.703**	0.59**	0.602**	-0.205**	-0.177**
Psychological health	-0.751**	0.67**	-0.65**	-0.337**	-0.283**	-0.375**	0.902**	0.703**	1	0.691**	0.677**	-0.141**	-0.159**
Social relationships	-0.579**	0. 538**	-0.483**	-0.245**	-0.186**	-0.335**	0.79**	0.59**	0.691**	1	0.602**	-0.12**	-0.119**
Environmental health	-0.546**	0.556**	-0.413**	-0.28**	-0.216**	-0.371**	0.861**	0.602**	0.677**	0.602**	1	-0.164**	-0.192**
Contamination obsessions	0.127**	-0.071	0.147**	0.334**	0.328**	0.221**	-0.187**	-0.205**	-0.141**	-0.12**	-0.164**	1	0.634**
Washing compulsions	0.166**	-0.094*	0.19**	0.283**	0.281**	0.18**	-0.193**	-0.177**	-0.159**	-0.119**	-0.192**	0.634**	1

Results of Pearson correlation showed that there were significant negative relationships between the independent variables (health anxiety, perceived stress and obsessive-compulsive disorder) with quality of life and its dimensions.

The fit indices of the model are presented in Table 4.

Table 4. Model fit indices -Goodness of fit

RMSEA	AGFI	GFI	NFI	NNFI	CFI	CMIN/DF
0.075	0.921	0.958	0.954	0.945	0.964	4.098

In general, the model fit indices (Table 4) indicate that the model has appropriate fit.

The structural equation modeling can be seen below:

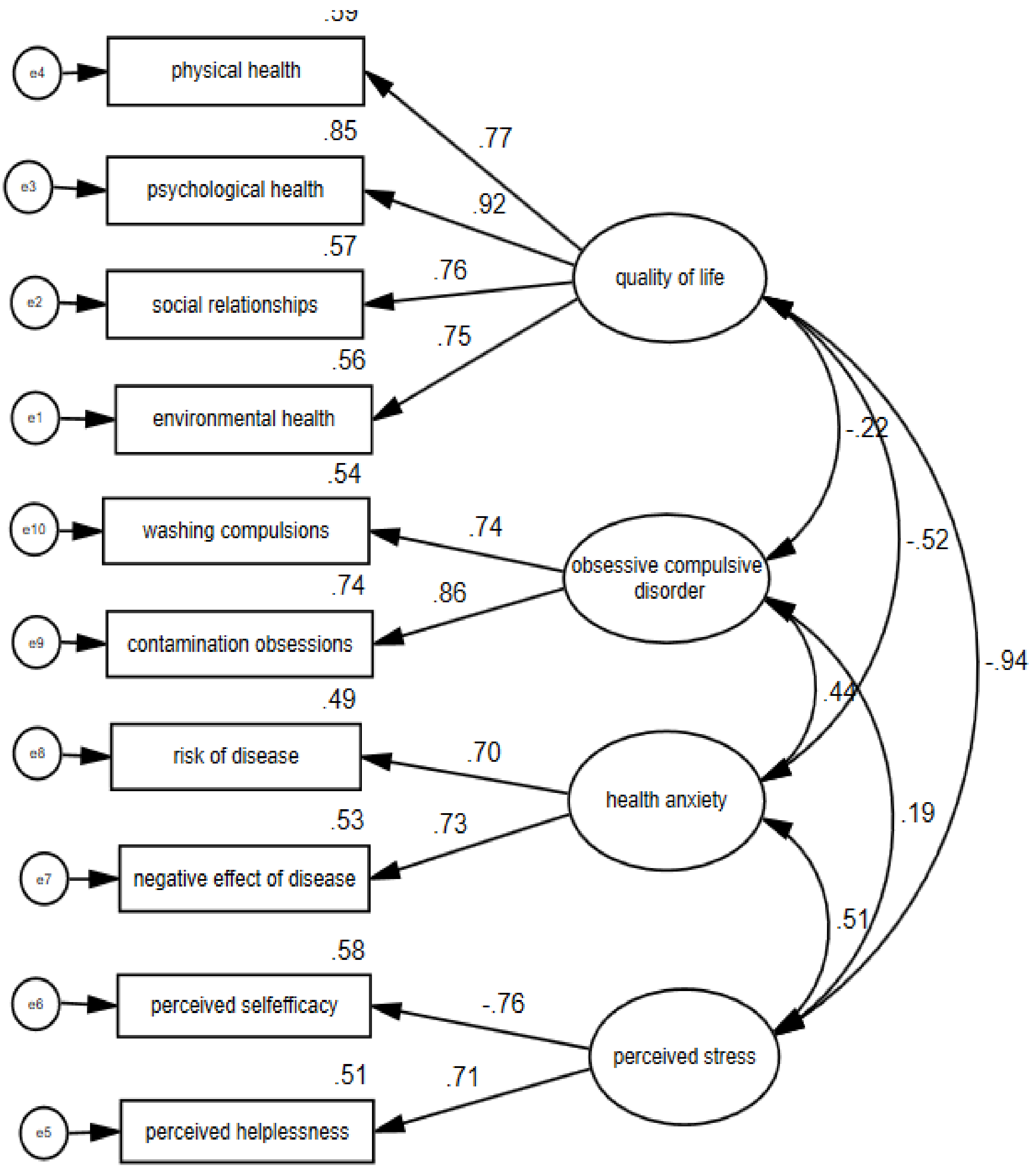

Fig 1. The structural equation modeling (SEM) of psychological problems during coronavirus

First, the regression model is fitted. The regression coefficients given in Table 5 indicate effectiveness or non-effectiveness of the subscales on the main variable. The subscales of environmental health with a standardized estimate of 0.747, social relationships with a standardized estimate of 0.757, psychological health with a standardized estimate of 0.923, and physical health with a standardized estimate of 0.767 correctly explain the quality of life variable. Perceived helplessness subscale with a standardized estimate of 0.713 and perceived

self-efficacy with a standardized estimate of -0.759 correctly explain the perceived stress variable. The subscale of negative effect of disease with a standardized estimate of 0.726 and risk of disease with a standardized estimate of 0.7 correctly explain the variable of health anxiety, and the subscale of contamination obsessions with standardized estimation of 0.861 and washing compulsions with a standardized estimate of 0.736 correctly explain the obsessive-compulsive variable.

Table 5. Regression Weight & Standardized Regression Weight

			Estimate	S.E.	C.R.	P	Standardized Estimate
environmental health	<---	Quality of life	1.000				.747
Social relationships	<---	Quality of life	.492	.027	18.061	<0.001	.757
Psychological health	<---	Quality of life	1.081	.049	22.118	<0.001	.923
Physical health	<---	Quality of life	.946	.052	18.324	<0.001	.767
Perceived helplessness	<---	Perceived stress	1.000				.713
Perceived self-efficacy	<---	Perceived stress	-.914	.056	-16.265	<0.001	-.759
Negative effect of disease	<---	Health anxiety	1.000				.726
Risk of disease	<---	Health anxiety	3.010	.301	10.014	<0.001	.700
Contamination obsessions	<---	obsessive compulsive disorder	1.000				.861
Washing compulsions	<---	obsessive compulsive disorder	.700	.088	7.96	<0.001	.736

Table 6. Covariances & Correlations

			Estimate	S.E.	C.R.	P	Correlations
Quality of life	<-->	obsessive compulsive disorder	-4.121	.961	-4.289	<0.001	-.222
Quality of life	<-->	Health anxiety	-3.038	.384	-7.903	<0.001	-.524
Perceived stress	<-->	Health anxiety	2.953	.412	7.175	<0.001	.513
Quality of life	<-->	Perceived stress	-14.097	1.228	-11.480	<0.001	-.941
Perceived stress	<-->	obsessive compulsive disorder	3.500	1.060	3.303	<0.001	.190
Health anxiety	<-->	obsessive compulsive disorder	3.126	.468	6.678	<0.001	.439

As seen in Table 6, there is a significant correlation between all variables in pairs. There is a negative correlation between obsessive-compulsive disorder and quality of life (r=-0.222), so that with increasing obsessive-compulsive disorder, the quality of life decreases and vice versa. There is a negative correlation between health anxiety and quality of life (r=-0.524). There is a positive correlation between health anxiety and perceived stress (r=0.513). There is a negative correlation between perceived stress and quality of life (r=-0.941). There is a positive correlation between obsessive-compulsive disorder and perceived stress (r=0.19) and finally, there is a positive correlation between obsessive-compulsive disorder and health anxiety (r=0.439).

Discussion

The present study assessed the quality of life and its related psychological problems during coronavirus pandemic. The results indicated that there are significant negative correlations between health anxiety, perceived stress and obsessive-compulsive disorder with quality of life.

The correlation between health anxiety and quality of life

The results showed that there is a negative correlation between health anxiety and quality of life. The current finding is consistent with findings from other studies conducted by Abdelghani et al [19], Hayter et al [32], Trougakos et al [33], Korkmaz et al [34], and Heidari Shams et al [35]. Trougakos et al. [33] indicated that Covid-19 health anxiety impair health (somatic complaints), home (family engagement), and job (goal progress) outcomes due to increased emotion suppression and lack of psychological need fulfillment. In addition, people who constantly engage in handwashing behavior were buffered from the negative effects of Covid-19 health anxiety. Korkmaz et al. [34] found that the quality of life in health care workers who worked in the Covid-19 outpatient clinics or emergency departments, decreases due to

increased levels of anxiety. Also, in Hayter et al's [32] study, the high health anxiety group reported poorer quality of life relative to the groups with relatively high or low health anxiety. In explaining this finding, it can be stated that the excessive increase of health anxiety in some people during the epidemic of viral diseases such as Covid-19 weakens the body's immune system against the disease and can affect the rational decisions and social behaviors of individuals. High levels of health anxiety are associated with catastrophizing changes in the body and the occurrence of maladaptive coping behaviors, leading to distress, social disability, occupational dysfunction, and frequent visits to health centers [36], resulting in reduced quality of life.

The correlation between health anxiety and obsessive-compulsive disorder

The results showed that there is a positive correlation between health anxiety and obsessive-compulsive disorder. This finding is consistent with previous studies conducted by Abdelghani et al. [19], Sica et al.[37], and Denns et al [38]. Abdelghani et al. [19] indicated that the psychological dimensions associated with health anxiety to Covid-19 infection were obsessive-compulsive disorder, depression, phobia, somatization, psychoticism and hostility. Sica et al. [37] in their study also found that after controlling for demographic variables and other internalizing problems, health anxiety was the most important factor associated with perceived dangerousness of Covid-19. The results of this study revealed that in addition to health anxiety, people with washing and contamination obsession may also be susceptible and vulnerable to Covid-19 fears. These individuals may be at risk of exacerbation of obsessive-compulsive symptoms during Covid-19. In explaining this finding, it can be stated that Individuals with pre-existing anxiety-related disorders, including obsessive-compulsive disorder (OCD), may be susceptible to developing excessive maladaptive anxiety during an outbreak of a pandemic. Compulsive washing and contamination fear are among the most common symptoms of OCD, and threats from epidemics for patients with OCD is highlighted by these symptoms. Also,

individuals with health anxiety perceive physical sensations such as coughing or muscle aches as symptoms of being sick. They compulsively check their body temperature to make sure that they are not sick. Individuals with severe health anxiety and OCD overestimate the likelihood of threat, which leads to increased levels of anxiety and often triggers checking behaviors [38]. In other words, people with high health anxiety engage in a variety of maladaptive safety behaviors, including obsessive hand washing, social isolation, and shopping with panic. In general, obsessive thoughts cause anxiety, while obsessive action related to it, is performed to reduce anxiety [39].

The correlation between perceived stress and quality of life

The results revealed a negative correlation between perceived stress and quality of life. This finding is consistent with previous studies conducted by Çelmeçe & Menekay [40], Tejoyuwono et al. [41], and Qi et al. [13]. Studies suggest that perceived stress leads to higher levels of anxiety and lower levels of health-related quality of life [42]. The results of a study conducted by Çelmeçe & Menekay [40] on healthcare professionals caring for Covid-19 patients, showed that there was a high and negative correlation between stress and quality of life. Tejoyuwono et al. [41] found that the students' mental health status (anxiety, depression and stress) during the pandemic coronavirus was dominantly on the normal category and the quality of life was moderate in all domains. Stress also significantly affected all domains of quality of life. In explaining this finding, it can be stated that prolonged quarantine, fear of disease, mental fatigue, insufficient information, financial loss, loss of freedom, uncertainty about the status of disease, uncertainty about time of disease control and the seriousness of the risk are among the stressors, which may affect the people's quality of life during this pandemic. On the other hand, when the situation is stressful and people fail to control the environmental stimuli, stress can negatively affect their well-being or performance.

The correlation between perceived stress and health anxiety

The results showed that there is a positive correlation between perceived stress and health anxiety. The results of a study conducted by Garboczy et al. [43] among university students amid the Covid-19 pandemic showed that there was a significant positive relationship between perceived stress and health anxiety. In such situations, there is a tendency to use coping mechanisms other than appropriate mechanisms, which in turn can lead to an increase in perceived stress levels [43]. Another study conducted in the UK during the first peak of the Covid 19 pandemic showed that community cohesion negatively predicted perceived stress and health anxiety. It also showed a strong positive association between health anxiety and perceived stress. Stress and health anxiety scores reached the peak level in the first weeks of the imposed quarantine. With extending the quarantine, participants reported lower scores on stress, health anxiety, and community cohesion and the positive association between stress and health anxiety was stronger among males during the quarantine [44]. Similar conditions were observed during the H1N1 influenza outbreak, and anxious individuals experienced higher levels of stress during the H1N1 influenza pandemic [45]. In situations such as epidemics, where there are many psychological challenges (such as worrying about essentials such as food availability and inability to plan long-term life), people with high stress experience higher anxiety [46].

The correlation between perceived stress and obsessive-compulsive disorder

The results also revealed a posiotive correlation between perceived stress and obsessive compulsive-disorder. Studies have indicated that the prevalence of perceived stress, anxiety, depression, and obsessive-compulsive symptoms increased significantly during the Covid-19 epidemic [47]. Kracker Imthon et al. [48] found that of the 1001 OCD patients, 605 (60.5%) reported experienceing at least one stressful life event during their lifetime. These results suggest that stressful life events may make Brazilian OCD patients more vulnerable to the onset

or exacerbation of obsessive-compulsive symptoms [48]. The results of a study conducted by ElliKoumantarou Malisiova on obsessive-compulsive patients also showed this correlation [49]. Obsessive Compulsive Disorder (OCD) may be the result of an interaction between genetic and environmental risk factors, including exposure to stressful and life-threatening events such as age-related changes in the reproductive systems, parenting styles, social and economic problems [50, 51]. Also, increased general stress (for example, job loss and family disease) and changes in normal life routines are among the causes of severe obsessive-compulsive disorder [48], and it is true about Covid-19 and the quarantine caused by it. The stress caused by the Covid-19 virus has caused many problems for people who are vulnerable to obsessive-compulsive disorder, as this behavior disrupts the normal course of their lives [52].

The correlation between obsessive-compulsive disorder and quality of life

The results showed a negative correlation between obsessive-compulsive disorder and quality of life. In previous epidemics such as Acute Respiratory Syndrome (SARS), Middle East Respiratory Syndrome (MERS), and the influenza, exacerbations of OCD have been reported 6 to 12 months after its outbreak. When coping strategies involved repetitive behaviors, there is a risk of increasing obsessive-compulsive disorder [52]. Shaoo et al. [53] found that patients with OCD scored significantly less in total score as well as all the domains of quality of life compared to healthy control group subjects. The main characteristic of obsessive-compulsive disorder (OCD) is the presence of recurrent or severe obsessions or compulsions that cause considerable suffering for person. These obsessions cause significant disruption in the normal process of life, normal social activities, and relationships [54] and thus negatively affect a person's quality of life. OCD as a disorder affects the quality of life in domains of mental health such as social functioning and role limitation due to emotional problems and mental health [55].

Study limitations

The first limitation of this study is that we selected participants to study through an online survey platform, those who do not have access to online surveys (e.g., the elderly), people who do not have access to the internet and people with low literacy, entered the study less than others and the findings may be prone to selection bias. Second, psychological health was reported by the participants themselves, and no acceptable evaluation tool was used. Third, the data in this study were self-reported, and participants' responses may be prone to social desirability bias. Therefore, future studies are suggested to design and implement intervention studies in the form of some models and theories such as social cognitive theory, protection motivation theory and stress management theory. Also, this study is recommended in other populations (such as patients recuperated from Covid-19 or individuals who have lost a family member due to Covid-19 disease) and with other psychological variables (such as depression, Post traumatic stress disorder (PTSD) or prolonged grief disorder).

Study strengths

-Data collection from 2 provinces of Iran (a city with commercial ports and an industrial city and a city with a lot of tourist attractions).

-Using the validated questionnaires

-Synchronization time coincides with pandemic peak time

Conclusion

The present findings show that the outbreak of coronavirus has changed people's living conditions and has had devastating psychological effects such as stress, health anxiety and obsessive behaviors, resulting in reduced quality of life. Thus, the design of psychological interventions to improve the mental health of individuals during the disease has a particular

importance and the necessary measures should be taken by policy makers and relevant officials in this regard. It is necessary to implement counseling and psychological interventions at the community level in the current situation, interventions such as: creating support networks with the presence of a counselor and a psychologist, telephone counseling, psychological support for medical staff, patients recuperated from Covid-19 or individuals who have lost a family member due to Covid-19 disease and so on.

Acknowledgements

The authors would like to thank all the participants in the study.

References

1. Bernard I, Limonta D, Mahal LK, Hobman TC. Endothelium infection and dysregulation by SARS-CoV-2: Evidence and caveats in COVID-19. Viruses. 2021;13(1):29.
2. Wu Z, McGoogan JM. Characteristics of and important lessons from the coronavirus disease 2019 (COVID-19) outbreak in China: summary of a report of 72 314 cases from the Chinese Center for Disease Control and Prevention. Jama. 2020;323(13):1239-42.
3. Moradzadeh R. The challenges and considerations of community-based preparedness at the onset of COVID-19 outbreak in Iran, 2020. Epidemiology & Infection. 2020;148.
4. WHO. WHO Coronavirus Disease (COVID-19) Dashboard Data 2021 .
5. Alizadehfard S, Alipuor A. The path analysis model in prediction of corona phobia based on intolerance of uncertainly and health anxiety. Journal of Research in Psychological Health. 2020;14(1):16-27.
6. Brooks SK, Webster RK, Smith LE, Woodland L, Wessely S, Greenberg N, et al. The psychological impact of quarantine and how to reduce it: rapid review of the evidence. The lancet. 2020;395(10227):912-20.

7. Wang Y, Xu B, Zhao G, Cao R, He X, Fu S. Is quarantine related to immediate negative psychological consequences during the 2009 H1N1 epidemic? General hospital psychiatry. 2011;33(1):75-7.

8. Rubin GJ, Wessely S. The psychological effects of quarantining a city. Bmj. 2020;368.

9. van Ballegooijen H, Goossens L, Bruin RH, Michels R, Krol M. Concerns, quality of life, access to care and productivity of the general population during the first 8 weeks of the coronavirus lockdown in Belgium and the Netherlands. BMC health services research. 2021;21(1):1-8.

10. Zhang W-r, Wang K, Yin L, Zhao W-f, Xue Q, Peng M, et al. Mental health and psychosocial problems of medical health workers during the COVID-19 epidemic in China. Psychotherapy and psychosomatics. 2020;89(4):242-50.

11. Shahyad S, Mohammadi MT. Psychological impacts of Covid-19 outbreak on mental health status of society individuals: a narrative review. Journal of Military Medicine. 2020;22(2):184-92.

12. Zarabadipour M, Asgari Ghonche MR, Asgari Ghonche S, Mirzadeh M. Psychological evaluation of the factors affecting the stress caused by COVID-19 outbreak in the medical staff and the community of Qazvin, Iran Spring 2020. Journal Mil Med. 2020;22(6):517-25.

13. Qi M, Li P, Moyle W, Weeks B, Jones C. Physical activity, health-related quality of life, and stress among the Chinese adult population during the COVID-19 pandemic. International journal of environmental research and public health. 2020;17(18):6494.

14. Asmundson GJ, Abramowitz JS, Richter AA, Whedon M. Health anxiety: current perspectives and future directions. Current psychiatry reports. 2010;12(4):306-12.

15. Kosic A, Lindholm P, Järvholm K, Hedman-Lagerlöf E, Axelsson E. Three decades of increase in health anxiety: Systematic review and meta-analysis of birth cohort changes in university student samples from 1985 to 2017. Journal of Anxiety Disorders. 2020;71:102208.

16. Asmundson GJ, Taylor S. How health anxiety influences responses to viral outbreaks like COVID-19: What all decision-makers, health authorities, and health care professionals need to know. Journal of anxiety disorders. 2020;71:102211.

17. Taylor S. The psychology of pandemics: Preparing for the next global outbreak of infectious disease: Cambridge Scholars Publishing; 2019.

18. M F. Psychological consequences of coronavirus outbreak in the community. Social Impact Assessment. 2020;1(2):207-25.

19. Abdelghani M, Mahdy R, El-Gohari H. Health anxiety to COVID-19 virus infection and its relationship to quality of life in a sample of health care workers in Egypt: a cross-sectional study. Archives of Psychiatry and Psychotherapy. 2021;23(1):19-28.

20. Seçer İ, Ulaş S. An investigation of the effect of COVID-19 on OCD in youth in the context of emotional reactivity, experiential avoidance, depression and anxiety. International journal of mental health and addiction. 2020:1-14.

21. Abramowitz JS, Jacoby RJ. Obsessive-compulsive disorder in the DSM-5. Clinical Psychology: Science and Practice. 2014;21(3):221.

22. Shafran R, Coughtrey A, Whittal M. Recognising and addressing the impact of COVID-19 on obsessive-compulsive disorder. The Lancet Psychiatry. 2020;7(7):570-2.

23. Abba-Aji A, Li, D., Hrabok, M., Shalaby, R., Gusnowski, A., Vuong, W., ... & Agyapong, V. I. COVID-19 pandemic and mental health: prevalence and correlates of new-onset obsessive-compulsive symptoms in a Canadian province. International Journal of Environmental Research and Public Health. 2020;17(19):6986.

24. Alimohammadi F, Setodeh-asl N, Karami A. Designing a model of quality of life in elderly based on perceived stress and tolerance of distress. Journal of Health and Care. 2019;21(1):53-65.

25. Salkovskis PM, Rimes KA, Warwick H, Clark D. The Health Anxiety Inventory: development and validation of scales for the measurement of health anxiety and hypochondriasis. Psychological medicine. 2002;32(5):843-53.

26. Abramowitz JS, Olatunji BO, Deacon BJ. Health anxiety, hypochondriasis, and the anxiety disorders. Behavior Therapy. 2007;38(1):86-94.

27. Cohen S, Kamarck T, Mermelstein R. A global measure of perceived stress. Journal of health and social behavior. 1983:385-96.

28. Taghavi SM, Mokarami H, Nazifi M, Choobineh A, Marioryad H, Kujerdi MF. The influence of socio-demographic, health and work-related factors on health-related quality of life among Iranian industrial workers. Health. 2014;2014.

29. Usefy A, Ghassemi GR, Sarrafzadegan N, Mallik S, Baghaei A, Rabiei K. Psychometric properties of the WHOQOL-BREF in an Iranian adult sample. Community mental health journal. 2010;46(2):139-47.

30. Sanavio E. Obsessions and compulsions: the Padua Inventory. Behaviour research and therapy. 1988;26(2):169-77.

31. Van Oppen P. Obsessions and compulsions: dimensional structure, reliability, convergent and divergent validity of the Padua Inventory. Behaviour Research and Therapy. 1992;30(6):631-7.

32. Hayter AL, Salkovskis PM, Silber E, Morris RG. The impact of health anxiety in patients with relapsing remitting multiple sclerosis: Misperception, misattribution and quality of life. British Journal of Clinical Psychology. 2016;55(4):371-86.

33. Trougakos JP, Chawla N, McCarthy JM. Working in a pandemic: Exploring the impact of COVID-19 health anxiety on work, family, and health outcomes. Journal of Applied Psychology. 2020.

34. Korkmaz S, Kazgan A, Çekiç S, Tartar AS, Balcı HN, Atmaca M. The anxiety levels, quality of sleep and life and problem-solving skills in healthcare workers employed in COVID-19 services. Journal of Clinical Neuroscience. 2020;80:131-6.

35. Shams JH, Mohammadzadeh KA, Maher A. Correlation between Health Literacy and quality of life with health anxiety in outpatient patients referred to Shahid Beheshti Specialized Polyclinic in Karaj. J Health Promot Manag. 2020;9(1):1-9.

36. Taylor S, Asmundson GJ, Hyprochondria. Treating health anxiety: A cognitive-behavioral approach: Guilford Press New York; 2004.

37. Sica C, Caudek C, Cerea S, Colpizzi I, Caruso M, Giulini P, et al. Health anxiety predicts the perceived dangerousness of covid-19 over and above intrusive illness-related thoughts, contamination symptoms, and state and trait negative affect. International Journal of Environmental Research and Public Health. 2021;18(4):1933.

38. Dennis D, Radnitz C, Wheaton MG. A Perfect Storm? Health Anxiety, Contamination Fears, and COVID-19: Lessons Learned from Past Pandemics and Current Challenges. International journal of cognitive therapy. 2021:1-17.

39. Piacentini J, Langley AK. Cognitive-behavioral therapy for children who have obsessive-compulsive disorder. Journal of clinical psychology. 2004;60(11):1181-94.

40. Çelmeçe N, Menekay M. The effect of stress, anxiety and burnout levels of healthcare professionals caring for COVID-19 patients on their quality of life. Frontiers in psychology. 2020;11:3329.

41. Tejoyuwono AAT, Nugraha RP, Fahdi FK. The Effect of Mental Health Status on the Quality of Life of Faculty of Medicine Students during the Pandemic Coronavirus Disease 2019 Period. Open Access Macedonian Journal of Medical Sciences. 2021;9(E):645-52.

42. Racic M, Todorovic R, Ivkovic N, Masic S, Joksimovic B, Kulic M. Self-perceived stress in relation to anxiety, depression and health-related quality of life among health

professions students: a cross-sectional study from Bosnia and Herzegovina. Slovenian Journal of Public Health. 2017;56(4):251.

43. Garbóczy S, Szemán-Nagy A, Ahmad MS, Harsányi S, Ocsenás D, Rekenyi V, et al. Health anxiety, perceived stress, and coping styles in the shadow of the COVID-19. BMC psychology. 2021;9(1):1-13.

44. Svensson SJ, Elntib S. Community cohesion during the first peak of the COVID-19 pandemic: A social antidote to health anxiety and stress. Journal of Applied Social Psychology. 2021.

45. Wheaton MG, Abramowitz JS, Berman NC, Fabricant LE, Olatunji BO. Psychological predictors of anxiety in response to the H1N1 (swine flu) pandemic. Cognitive Therapy and Research. 2012;36(3):210-8.

46. Office for National Statistics. Coronavirus and the social impacts on Great Britain. 2020.

47. Mrklas K, Shalaby R, Hrabok M, Gusnowski A, Vuong W, Surood S, et al. Prevalence of perceived stress, anxiety, depression, and obsessive-compulsive symptoms in health care workers and other workers in Alberta during the COVID-19 pandemic: cross-sectional survey. JMIR Mental Health. 2020;7(9):e22408.

48. Kracker Imthon A, Antônio Caldart C, do Rosário MC, Fontenelle LF, Constantino Miguel E, Arzeno Ferrão Y. Stressful life events and the clinical expression of Obsessive–Compulsive Disorder (OCD): an exploratory study. Journal of clinical medicine. 2020;9(10):3371.

49. Malisiova EK, Mourikis I, Chalimourdas T, Nianiakas N, Michou M, Mantzou A, et al. Low hair cortisol concentrations in obsessive compulsive disorder: a cross-sectional study. Journal of Psychiatric Research. 2020;131:187-93.

50. Adams TG, Kelmendi B, Brake CA, Gruner P, Badour CL, Pittenger C. The role of stress in the pathogenesis and maintenance of obsessive-compulsive disorder. Chronic Stress. 2018;2:2470547018758043.

51. Brander G, Perez-Vigil A, Larsson H, Mataix-Cols D. Systematic review of environmental risk factors for obsessive-compulsive disorder: a proposed roadmap from association to causation. Neuroscience & Biobehavioral Reviews. 2016;65:36-62.

52. Banerjee D. The other side of COVID-19: Impact on obsessive compulsive disorder (OCD) and hoarding. Psychiatry research. 2020;288:112966.

53. Sahoo P, Sethy RR, Ram D. Functional impairment and quality of life in patients with obsessive compulsive disorder. Indian journal of psychological medicine. 2017;39(6):760-5.

54. Kaplan HI, Sadock BJ. Synopsis of psychiatry: Behavioral sciences clinical psychiatry: Williams & Wilkins Co; 1988.

55. Koran LM, Thienemann ML, Davenport R. Quality of life for patients with obsessive-compulsive disorder. The American journal of psychiatry. 1996.

SPSS file (data)

Click here to access/download
Supporting Information
totaldata.sav

Figure

Click here to access/download
Supporting Information
Fig1.tif

Quality of life and its related psychological problems during coronavirus pandemic

Psychological problems during coronavirus

Atefeh Homayuni[1], Zahra Hosseini[2]*, Nahid Shahabi[3], Roghayeh Ezati Rad[4], Farah Moayyedi[5]

1.Student Research Committee, Hormozgan University of Medical Sciences, Bandar Abbas, Iran.
2. Tobacco and Health Research Center, Hormozgan University of Medical Sciences, Bandar Abbas, Iran.
3. Student Research Committee, Hormozgan University of Medical Sciences, Bandar Abbas, Iran.
4. Student Research Committee, Hormozgan University of Medical Sciences, Bandar Abbas, Iran.
5. Tobacco and Health Research Center, Hormozgan University of Medical Sciences, Bandar Abbas, Iran.

***Corresponding Author**

Email: hosseinishirin@ymail.com (ZH)

Abstract

Background: The prevalence of coronavirus disease 2019 (COVID-19) has endangered the psychological health of individuals. This study aimed to assess the quality of life and its related psychological problems during coronavirus pandemic.

Methods: In this cross-sectional study, 559 citizens above the age of 16 years, in Isfahan and Bandar Abbas cities in Iran were selected with a convenient sampling method. An online questionnaire was used to collect the data, which consisted of five sections: demographic information, short health anxiety inventory (SHAI), perceived stress scale (PSS), world health organization quality of life questionnaire (WHOQOL-BREF) and Padua inventory. Data were analyzed using statistical tests including t-test, path analysis and structural equation modeling (SEM) using SPSS 24 and Amos 21 statistical software.

Results: There were significant positive relationships between health anxiety and perceived stress (r=0/513), obsessive-compulsive disorder and perceived stress (r=0/19), obsessive-compulsive disorder and health anxiety (r=0/439). Also there were significant negative relationships between obsessive-compulsive disorder and quality of life (r=-0/222), health anxiety and quality of life (r=-0/524), perceived stress and quality of life (r=-0/941).

Conclusions: According to our findings, health anxiety, perceived stress and obsessive-compulsive disorder are among the most common psychological problems during coronavirus pandemic which affects the quality of life. Therefore, prevention and treatment of these psychological consequences is one of the most important priorities in the global crisis of Covid-19 disease.

Keywords: Contamination Obsessions, Coronavirus Pandemic, Health Anxiety, Perceived Stress, Quality of Life, Washing Compulsions

Introduction

Coronavirus disease (Covid-19) pandemic was first seen in December 2019 in china. The virus spread rapidly around the world and it was recognized as a pandemic by the World Health Organization in March 2020 [1]. Most symptoms of the Covid-19 include fever, cough, shortness of breath, and gastrointestinal symptoms in some cases. In more severe cases, it may cause Acute Respiratory Distress Syndrome (ARDS) and hypoxic respiratory failure, which is a major cause of death in Covid-19 patients [2].

The first case of this disease in Iran was diagnosed in February 19, 2020, when two cases of Covid-19 were reported in Qom, and then the disease spread throughout the Iran [3]. Based on the World Health Organization, up to September 13, 2021, 5237799 cases were confirmed and a total of 112935 deaths have been confirmed in Iran [4].

Covid-19 has imposed a serious threat to physical health and life and has caused a wide range of psychological and physical problems. Some factors such as fear of being infected with the virus, fear of death, spreading false news and rumors, interference in daily activities, travel prohibitions or restrictions, reduced social relationships (colleagues, friends and family), job and financial problems, and several factors related to these conditions endangered the mental health of people [5]. Thus, the issue of psychological effects of this viral disease on the mental health of people at different levels of society is crucial. All studies, examined the psychological disorders of people in quarantine, have reported many signs of mental damage such as emotional disorder, depression, stress, irritability, insomnia, decreased attention, post-traumatic stress disorder, anger and emotional blunting [6-8].

The Covid-19 pandemic has had great disruptive impacts on communities throughout the world due to fear and stress. These fears include fears for unemployment due to Covid 19-related restrictions, financial worries, and health-related worries. These stresses and worries

may reduce the quality of life [9]. The Covid-19 pandemic has also been associated with impaired health-related quality of life among local residents in China [10].

The most common triggers of psychological disorders and stress in people quarantined due to Covid-19 disease have been reported to be fear of being infected or infecting others, long period of quarantine, inadequate support and lack of access to care adequate medicine and food, and ultimately fatigue and boredom due to quarantine and isolation [11]. Zarabadipour et al. showed that access to social response systems, considerable weight change and reluctance to perform daily activities were among the factors, which had a significant relationship with stress scores in the medical staff during Covid-19 pandemic. In this study, gender, the presence of high risk elderly person in the family, having a fatal case due to the Covid-19 in the family, exacerbation of the chronic disease, job closure, etc. had significant relationships with stress score [12]. Qi et al. [13] in their study among Chinese adults during the Covid-19 pandemic reported that perceived stress scores were negatively correlated with physical component summary score (r=-0.4) and mental component summary score for health related quality of life (r=-0.6). It indicates that participants who reported higher health-related quality of life, experienced lower levels of perceived stress.

Health anxiety occurs when mild physical changes are interpreted as a sign of a disease (usually a serious and dangerous disease) [14]. Most people experience some degree of health anxiety. This awareness can be a protective factor against health threatening factors and play a major role in identifying the early symptoms of the health issue and enhancing health-promoting behaviors [15]. However, its excessive level can be dangerous and harmful to the person [14]. Recent studies in coronavirus pandemics have shown that the frequency and severity of health anxiety have increased significantly [16]. In the case of infectious diseases or pandemic conditions, people with high health anxiety are prone to misunderstanding and they consider all these changes as evidence for an infectious disease [17]. People with high health anxiety

also engage in a variety of non-adaptive safety behaviors. During this disease outbreak, it may be followed by over-washing of hands, social isolation, and purchasing with fear. Low levels of health anxiety can also have negative effects on health behavior. People who find themselves at low risk for disease are also less likely to change their social behavior and ignore recommendations for social distance. Thus, health anxiety is one of several psychological factors that affect one's way of reaction to the spread of the coronavirus [18]. The results of a cross-sectional study conducted by Abdelghani et al. [19] on health care workers in hospitals of Egypt showed that health anxiety to Covid-19 virus was inversely correlated with all domains of quality of life (physical health, r=-0.409; psychological health, r=-0.453; social relationships, r= -0.224 and environmental health, r=-0.385).

Disease-related obsessive-compulsive disorder is another common psychological disorder during the epidemic. Information provided by various sources about the methods of infection with the virus may increase people fear of this disease infection. Fear is a defense mechanism that a person shows against dangerous situations and includes the basic reactions necessary to survive from these threatening situations. However, fear that is not appropriate to the current situation may lead to a variety of psychological disorders such as obsessive-compulsive disorder, anxiety, depression and panic [20]. According to the American Psychological Association, fear of contamination is the most common obsession. Fear of contamination is often associated with mandatory rituals such as hand washing, cleaning, and taking appropriate action to reduce exposure to contaminated sources [21]. Since observing personal hygiene (regular hand washing, use of masks and gloves) and maintaining social distance are among the most important ways to prevent the spread of Covid-19 virus, the risk of practical obsessions during the Coronavirus epidemic are considered very likely [22]. 60.3% of participants in the Abba-Aji et al.'s study [23] reported onset of obsessive-compulsive disorder symptoms (obsessions related to contamination with dirt, germs or viruses) and 53.8% had

compulsions to wash hands repeatedly or in a special way, during the Covid-19 pandemic. Respondents who showed obsessive-compulsive disorder symptoms only since the start of Covid-19 pandemic were significantly more likely to have moderate/high stress. Delay in starting vaccination, people's negligence in observing health protocols and their resistance to vaccination, led to higher prevalence and mortality due to the coronavirus in Iran, and as a result, imposed extra psychological pressure on people and decreased their quality of life. Residents of Hormozgan (a city with commercial ports) and Isahan (an industrial city and a city with a lot of tourist attractions) provinces are more vulnerable to coronavirus disease. Since, the first step in creating effective interventions to promote people's quality of life during coronavirus is to identify the relevant and predictive psychological factors, therefore, the present study was conducted to assess quality of life and its related psychological problems during coronavirus pandemic among people over 16 years old in Bandar Abbas and Isfahan.

Materials and methods

Study design and population

This descriptive-correlational study was conducted from October 22, 2020 to February 3, 2021. The statistical population of this study consisted of residents over 16 years old in Isfahan and Bandar Abbas (in Iran).

Sample size and sampling procedure

The sample size was calculated based on the following formula:

$$n = \left[(z_{1-\alpha/2} + z_{1-\beta})/C\right]^2 + 3 \ , \ C = 0.5 * \ln[(1+r)/(1-r)]$$

Assuming a 5% error, 90% test power and a correlation coefficient of -0.18 in previous studies [24], the sample size was estimated at 320 people based on the formula. Considering the design effect of 1.2 for cluster sampling, the final sample size was estimated at 384.

The inclusion criteria for the sample selection included: literacy, access to the Internet to answer questions, not suffering from psychological disorders such as obsessive-compulsive disorder, the minimum age of 16 years, living in Isfahan or Bandar Abbas and willingness to participate in the study. Exclusion criteria also included people with psychological disorders such as obsessive-compulsive disorder and unwillingness to participate in the study.

Regarding to the existing limitations due to the outbreak of Covid-19 and the impossibility of distributing questionnaires in paper form, online questionnaires were designed to collect the data. Questionnaires were sent to Hormozgan and Isfahan provinces through various social media (WhatsApp, Telegram, Linkedin), email, channels and news agencies, public relations of University of Medical Sciences, Red Crescent, Municipality and University Student Research Committee. The questionnaire link was submitted to the participants via various online channels. After publishing the questionnaire link, the people who received it were asked to complete the questionnaire (if they wished) and send it to other people they know. Finally, the participants registered their answers by clicking the submit button. To emphasize on the greater participation of individuals in the study, messages and links to participate in the study were resent as a reminder two weeks after the first submission. Also, with the cooperation of health centers in the studied provinces, a questionnaire link was sent to all people covered by healthcare centers in villages and cities. On the first page of the questionnaire, the purpose of the study was clearly explained to the study participants and they were reminded that mentioning their first and last names would not be necessary and their information would be kept confidential by the research team.

Ethics statement

Ethical approval was received for this study from the Ethics Committee of the Hormozgan University of Medical Sciences (IR.HUMS.REC.1399.439). Written informed consent was

obtained from individuals who participated in this study. For participants between 16-18 years of old, the research ethics committee waived the requirement for parental consent.

Research tools

The following demographic questionnaire and self-administered tools were used to measure variables and collect data. The assessed demographic characteristics were gender, marital status, education level, age and employment status, history of infection with Covid-19, underlying diseases.

1- Short Health Anxiety Inventory (SHAI): This inventory is an 18-item self-report scale. Each of the 18 items consists of four statements, in which individuals select the one that best reflects their feelings during the last six months. Items assess worry about health, awareness of bodily sensations or changes, and feared consequences of having an illness. The responses are scored on a 4-point Likert scale (0-3). This questionnaire enables estimating the magnitude of health anxiety in two components: the probability of getting the disease (14 questions) and the negative consequences of getting the disease (4 questions). Overall scores ranged from 0 to 54, whereby the higher scores indicated the higher health anxiety [25]. Several studies have shown that this scale has good internal consistency, as its internal consistency coefficients ranged from 0.71 to 0.95. In the same studies, the reliability coefficient through test-retest method with a one-week interval has been obtained at 0.90 and it has been shown that this scale can distinguish hypochondriasis patients from non-clinical control groups [26]. In the present study, Cronbach's alpha coefficient for this questionnaire was 0.855.

Perceived Stress Scale (PSS): The Perceived Stress Scale is a 14-item self-report tool developed by Cohen et al. (1983) to measure general perceived stress, thoughts and feelings about stressful events, control, overcoming, coping with stress, and experienced stress over the past month. On this scale, individuals are asked to indicate their feelings over the past 4 weeks

on a 5-point Likert scale from 0 (never) to 4 (always). For example, "In the past month, how often have you felt that you were unable to control the important things in your life?" This scale measures two subscales: a) Perceived helplessness, which includes items 1, 2, 3, 8, 11, 12, and 14. B) Perceived self-efficacy, which includes items 4, 5, 6, 7, 9, 10, and 13, and these items are scored inversely. The lowest score is zero and the highest score is 56. The higher scores indicated the higher perceived stress. In a study conducted by Cohen et al. (1983), the internal consistency coefficients for each of the subscales and the general PSS were between 0.84 to 0.86 [27]. The reliability coefficients of the internal consistency for this scale were obtained 0.84 to 0.86 through Cronbach's alpha coefficient in two groups of students and one group of smokers in the smoking cessation program. Perceived stress scale is significantly correlated with life events, depressive and physical symptoms, social anxiety, and low life satisfaction [27]. In the present study, Cronbach's alpha coefficient for this questionnaire was 0.887.

World Health Organization Quality of Life Questionnaire (WHOQOL-BREF): The Persian version of the WHOQOL-BREF was used to collect information. This questionnaire is a 26-item instrument consisting of four domains: physical health (7 items), psychological health (6 items), social relationships (3 items) and environmental health (8 items). The first two questions assess the general health and quality of life. Each question is scored on a 5-point Likert scale (1: strongly disagree to 5: strongly agree). The score of each aspect is calculated separately from the total score of its questions, so that score 4 indicates the worst and score 20 indicates the best situation in that aspect [28]. In this study, the standardized version of this questionnaire (developed by Yousefy et al. [29]) was used. Cronbach's alpha coefficient for physical health, psychological health, social relationships and environmental health were 0.81, 0.72, 0.78, and 0.76, respectively. In the present study, Cronbach's alpha coefficient for this questionnaire was 0.932.

Washing and Contamination Obsessions Questionnaire: To measure washing compulsions and contamination obsessions, Padua Questionnaire (Modified by Washington State University) (Obsessive-Compulsive Disorder Scale) was used [30]. Subjects' responses to each item are scored on a 5-point Likert scale (0=not at all, 1=low, 2=somewhat, 3=high, and 4=very high). High scores indicated a high level of obsessive-compulsive disorder in the subject. Van Open [31] obtained obsessive-compulsive disorder coefficients at 0.94 for Padua questionnaire and above 0.80 for its subscales. In the present study, Cronbach's alpha coefficient for this questionnaire was 0.875.

Data Analysis

Data were analyzed in SPSS-24 and Amos-21 statistical software. Demographic characteristics were described using descriptive statistics including frequency and percentage. Mean, standard deviation, minimum and maximum values were calculated for continuous variables. To test the hypotheses and discover the relationships between the variables, t-test, path analysis and structural equation modeling (SEM) were used. The level of significance was considered to be 95% ($p < 0.05$).

Results

A total number of 559 questionnaires were completed and returned. The participants' demographic information is as follows: 21.5% of the participants were male and 78.5% were female. More than half of the participants were married (71.6%) and the rest were single (26.8%), divorced (1.1%) or widow (0.5%). The majority of them had bachelor's degree (42.9%). In relation to job situation, 40.9% of the participants were employees. Only 14.5% of the participants were members of the medical, health and treatment staff and 83.4% of them had no history of chronic diseases (Table 1).

Table 1. Participants' demographic information (N = 559)

Characteristics	Categories	n(%)
Gender	Male	120 (21.5)
	Female	439 (78.5)
Marital status	Single	150 (26.8)
	Married	400 (71.6)
	Widow	3 (0.5)
	Divorced	6 (1.1)
Level of education	High school	36 (6.5)
	Diploma	100 (17.9)
	Associate degree	43 (7.7)
	Bachelor's degree	240 (42.9)
	Master's degree and higher	140 (25)
Job situation	Student	20 (3.6)
	University student	39 (7)
	Unemployed	25 (4.5)
	Housewife	181 (32.4)
	Employee	229 (40.9)
	Self-employed	65 (11.6)
Smoking	Only cigarette	23 (4.1)
	Only hookah	22 (3.9)
	Both of them	5 (0.9)
	None of them	509 (91.1)
Medical staff	Yes	81 (14.5)
	No	478 (85.5)
Chronic diseases	Yes	93 (16.6)
	No	466 (83.4)

Descriptive statistics (mean, standard deviation, minimum and maximum values) of research variables are reported in Table 2.

Table 2. Descriptive statistics of research variables

	Minimum	Maximum	Mean	Std. Deviation
age	16	69	37.34	11.19
Quality of life	37.00	128.00	87.03	15.81
Physical health	8.00	35.00	24.35	4.80
Psychological health	7.00	30.00	19.15	4.56
Social relationships	3.00	15.00	10.01	2.53
Environmental health	11.00	40.00	26.26	5.21
Obsessive-compulsive disorder	.00	40.00	19.70	9.12
Contamination obsessions	.00	24.00	10.69	5.54
Washing compulsions	.00	16.00	9.01	4.53
Health anxiety	.00	47.00	14.15	7.67
Risk of disease	.00	40.00	12.32	6.42
Negative effect of disease	.00	12.00	1.83	2.06
Perceived stress	2.00	54.00	27.4955	8.83518
Perceived helplessness	.00	28.00	13.92	5.41

Perceived self-efficacy	.00	28.00	14.43	4.65

As the results showed, the mean and standard deviation scores of perceived stress were (27.49±8.83), health anxiety (14.15±7.67), obsessive-compulsive disorder (19.7±9.12) and quality of life (87.03±15.81).

The correlation matrix of the variables is presented in Table 3.

Table 3. correlation coefficients of study variables

Variable	1	2	3	4	5	6	7	8	9	10	11	12	13
Perceived stress	1	-0.857**	0.897**	0.338**	0.298**	0.332**	-0.715**	-0.569**	-0.751**	-0.579**	-0.546**	0.127**	0.166**
Perceived self-efficacy	-0.857**	1	-0.541**	-0.231**	-0.186**	-0.279**	0.665**	0.508**	0.67**	0.538**	0.556**	-0.071	-0.094*
Perceived helplessness	0.897**	-0.541**	1	0.355**	0.327**	0.303**	-0.597**	-0.493**	-0.65**	-0.483**	-0.413**	0.147**	0.19**
Health anxiety	0.338**	-0.231**	0.355**	1	0.973**	0.693**	-0.366**	-0.357**	-0.337**	-0.245**	-0.28**	0.334**	0.283**
Risk of disease	0.298**	-0.186**	0.327**	0.973**	1	0.508**	-0.302**	-0.316**	-0.283**	-0.186**	-0.216**	0.328**	0.281**
Negative effect of disease	0.322**	-0.279**	0.303**	0.693**	0.508**	1	-0.424**	-0.346**	-0.375**	-0.335**	-0.371**	0.221**	0.18**
Quality of life	-0.715**	0.665**	-0.597**	-0.366**	-0.302**	-0.424**	1	0.851**	0.902**	0.79**	0.861**	-0.187**	-0.193**
Physical health	-0.569**	0.508**	-0.493**	-0.357**	-0.316**	-0.346**	0.851**	1	0.703**	0.59**	0.602**	-0.205**	-0.177**
Psychological health	-0.751**	0.67**	-0.65**	-0.337**	-0.283**	-0.375**	0.902**	0.703**	1	0.691**	0.677**	-0.141**	-0.159**
Social relationships	-0.579**	0. 538**	-0.483**	-0.245**	-0.186**	-0.335**	0.79**	0.59**	0.691**	1	0.602**	-0.12**	-0.119**
Environmental health	-0.546**	0.556**	-0.413**	-0.28**	-0.216**	-0.371**	0.861**	0.602**	0.677**	0.602**	1	-0.164**	-0.192**
Contamination obsessions	0.127**	-0.071	0.147**	0.334**	0.328**	0.221**	-0.187**	-0.205**	-0.141**	-0.12**	-0.164**	1	0.634**
Washing compulsions	0.166**	-0.094*	0.19**	0.283**	0.281**	0.18**	-0.193**	-0.177**	-0.159**	-0.119**	-0.192**	0.634**	1

Results of Pearson correlation showed that there were significant negative relationships between the independent variables (health anxiety, perceived stress and obsessive-compulsive disorder) with quality of life and its dimensions.

The fit indices of the model are presented in Table 4.

Table 4. Model fit indices -Goodness of fit

RMSEA	AGFI	GFI	NFI	NNFI	CFI	CMIN/DF
0.075	0.921	0.958	0.954	0.945	0.964	4.098

In general, the model fit indices (Table 4) indicate that the model has appropriate fit.

The structural equation modeling can be seen below:

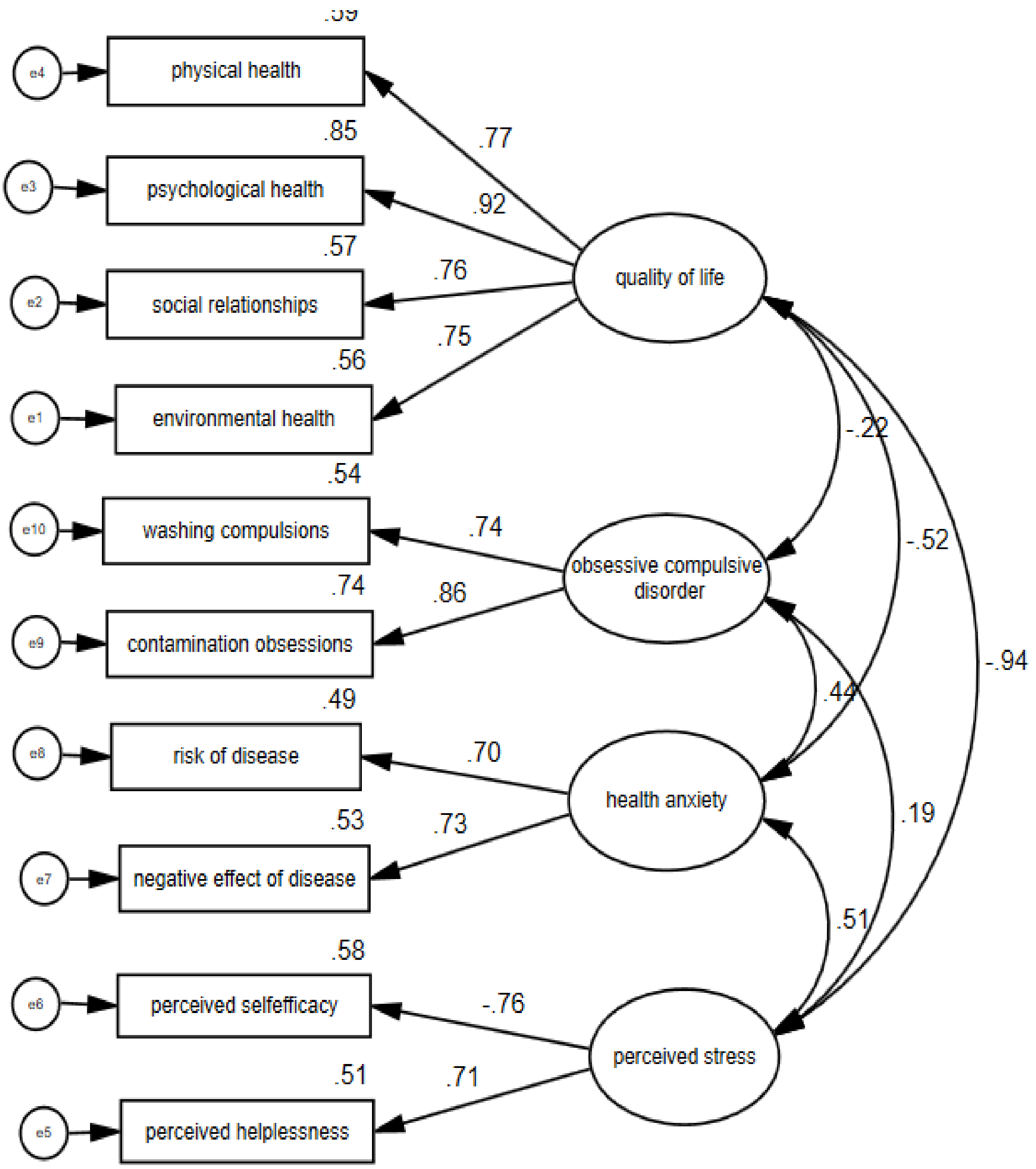

Fig 1. The structural equation modeling (SEM) of psychological problems during coronavirus

First, the regression model is fitted. The regression coefficients given in Table 5 indicate effectiveness or non-effectiveness of the subscales on the main variable. The subscales of environmental health with a standardized estimate of 0.747, social relationships with a standardized estimate of 0.757, psychological health with a standardized estimate of 0.923, and physical health with a standardized estimate of 0.767 correctly explain the quality of life variable. Perceived helplessness subscale with a standardized estimate of 0.713 and perceived

self-efficacy with a standardized estimate of -0.759 correctly explain the perceived stress variable. The subscale of negative effect of disease with a standardized estimate of 0.726 and risk of disease with a standardized estimate of 0.7 correctly explain the variable of health anxiety, and the subscale of contamination obsessions with standardized estimation of 0.861 and washing compulsions with a standardized estimate of 0.736 correctly explain the obsessive-compulsive variable.

Table 5. Regression Weight & Standardized Regression Weight

			Estimate	S.E.	C.R.	P	Standardized Estimate
environmental health	<---	Quality of life	1.000				.747
Social relationships	<---	Quality of life	.492	.027	18.061	<0.001	.757
Psychological health	<---	Quality of life	1.081	.049	22.118	<0.001	.923
Physical health	<---	Quality of life	.946	.052	18.324	<0.001	.767
Perceived helplessness	<---	Perceived stress	1.000				.713
Perceived self-efficacy	<---	Perceived stress	-.914	.056	-16.265	<0.001	-.759
Negative effect of disease	<---	Health anxiety	1.000				.726
Risk of disease	<---	Health anxiety	3.010	.301	10.014	<0.001	.700
Contamination obsessions	<---	obsessive compulsive disorder	1.000				.861
Washing compulsions	<---	obsessive compulsive disorder	.700	.088	7.96	<0.001	.736

Table 6. Covariances & Correlations

			Estimate	S.E.	C.R.	P	Correlations
Quality of life	<-->	obsessive compulsive disorder	-4.121	.961	-4.289	<0.001	-.222
Quality of life	<-->	Health anxiety	-3.038	.384	-7.903	<0.001	-.524
Perceived stress	<-->	Health anxiety	2.953	.412	7.175	<0.001	.513
Quality of life	<-->	Perceived stress	-14.097	1.228	-11.480	<0.001	-.941
Perceived stress	<-->	obsessive compulsive disorder	3.500	1.060	3.303	<0.001	.190
Health anxiety	<-->	obsessive compulsive disorder	3.126	.468	6.678	<0.001	.439

As seen in Table 6, there is a significant correlation between all variables in pairs. There is a negative correlation between obsessive-compulsive disorder and quality of life (r=-0.222), so that with increasing obsessive-compulsive disorder, the quality of life decreases and vice versa. There is a negative correlation between health anxiety and quality of life (r=-0.524). There is a positive correlation between health anxiety and perceived stress (r=0.513). There is a negative correlation between perceived stress and quality of life (r=-0.941). There is a positive correlation between obsessive-compulsive disorder and perceived stress (r=0.19) and finally, there is a positive correlation between obsessive-compulsive disorder and health anxiety (r=0.439).

Discussion

The present study assessed the quality of life and its related psychological problems during coronavirus pandemic. The results indicated that there are significant negative correlations between health anxiety, perceived stress and obsessive-compulsive disorder with quality of life.

The correlation between health anxiety and quality of life

The results showed that there is a negative correlation between health anxiety and quality of life. The current finding is consistent with findings from other studies conducted by Abdelghani et al [19], Hayter et al [32], Trougakos et al [33], Korkmaz et al [34], and Heidari Shams et al [35]. Trougakos et al. [33] indicated that Covid-19 health anxiety impair health (somatic complaints), home (family engagement), and job (goal progress) outcomes due to increased emotion suppression and lack of psychological need fulfillment. In addition, people who constantly engage in handwashing behavior were buffered from the negative effects of Covid-19 health anxiety. Korkmaz et al. [34] found that the quality of life in health care workers who worked in the Covid-19 outpatient clinics or emergency departments, decreases due to

increased levels of anxiety. Also, in Hayter et al's [32] study, the high health anxiety group reported poorer quality of life relative to the groups with relatively high or low health anxiety. In explaining this finding, it can be stated that the excessive increase of health anxiety in some people during the epidemic of viral diseases such as Covid-19 weakens the body's immune system against the disease and can affect the rational decisions and social behaviors of individuals. High levels of health anxiety are associated with catastrophizing changes in the body and the occurrence of maladaptive coping behaviors, leading to distress, social disability, occupational dysfunction, and frequent visits to health centers [36], resulting in reduced quality of life.

The correlation between health anxiety and obsessive-compulsive disorder

The results showed that there is a positive correlation between health anxiety and obsessive-compulsive disorder. This finding is consistent with previous studies conducted by Abdelghani et al. [19], Sica et al.[37], and Denns et al [38]. Abdelghani et al. [19] indicated that the psychological dimensions associated with health anxiety to Covid-19 infection were obsessive-compulsive disorder, depression, phobia, somatization, psychoticism and hostility. Sica et al. [37] in their study also found that after controlling for demographic variables and other internalizing problems, health anxiety was the most important factor associated with perceived dangerousness of Covid-19. The results of this study revealed that in addition to health anxiety, people with washing and contamination obsession may also be susceptible and vulnerable to Covid-19 fears. These individuals may be at risk of exacerbation of obsessive-compulsive symptoms during Covid-19. In explaining this finding, it can be stated that Individuals with pre-existing anxiety-related disorders, including obsessive-compulsive disorder (OCD), may be susceptible to developing excessive maladaptive anxiety during an outbreak of a pandemic. Compulsive washing and contamination fear are among the most common symptoms of OCD, and threats from epidemics for patients with OCD is highlighted by these symptoms. Also,

individuals with health anxiety perceive physical sensations such as coughing or muscle aches as symptoms of being sick. They compulsively check their body temperature to make sure that they are not sick. Individuals with severe health anxiety and OCD overestimate the likelihood of threat, which leads to increased levels of anxiety and often triggers checking behaviors [38]. In other words, people with high health anxiety engage in a variety of maladaptive safety behaviors, including obsessive hand washing, social isolation, and shopping with panic. In general, obsessive thoughts cause anxiety, while obsessive action related to it, is performed to reduce anxiety [39].

The correlation between perceived stress and quality of life

The results revealed a negative correlation between perceived stress and quality of life. This finding is consistent with previous studies conducted by Çelmeçe & Menekay [40], Tejoyuwono et al. [41], and Qi et al. [13]. Studies suggest that perceived stress leads to higher levels of anxiety and lower levels of health-related quality of life [42]. The results of a study conducted by Çelmeçe & Menekay [40] on healthcare professionals caring for Covid-19 patients, showed that there was a high and negative correlation between stress and quality of life. Tejoyuwono et al. [41] found that the students' mental health status (anxiety, depression and stress) during the pandemic coronavirus was dominantly on the normal category and the quality of life was moderate in all domains. Stress also significantly affected all domains of quality of life. In explaining this finding, it can be stated that prolonged quarantine, fear of disease, mental fatigue, insufficient information, financial loss, loss of freedom, uncertainty about the status of disease, uncertainty about time of disease control and the seriousness of the risk are among the stressors, which may affect the people's quality of life during this pandemic. On the other hand, when the situation is stressful and people fail to control the environmental stimuli, stress can negatively affect their well-being or performance.

The correlation between perceived stress and health anxiety

The results showed that there is a positive correlation between perceived stress and health anxiety. The results of a study conducted by Garboczy et al. [43] among university students amid the Covid-19 pandemic showed that there was a significant positive relationship between perceived stress and health anxiety. In such situations, there is a tendency to use coping mechanisms other than appropriate mechanisms, which in turn can lead to an increase in perceived stress levels [43]. Another study conducted in the UK during the first peak of the Covid 19 pandemic showed that community cohesion negatively predicted perceived stress and health anxiety. It also showed a strong positive association between health anxiety and perceived stress. Stress and health anxiety scores reached the peak level in the first weeks of the imposed quarantine. With extending the quarantine, participants reported lower scores on stress, health anxiety, and community cohesion and the positive association between stress and health anxiety was stronger among males during the quarantine [44]. Similar conditions were observed during the H1N1 influenza outbreak, and anxious individuals experienced higher levels of stress during the H1N1 influenza pandemic [45]. In situations such as epidemics, where there are many psychological challenges (such as worrying about essentials such as food availability and inability to plan long-term life), people with high stress experience higher anxiety [46].

The correlation between perceived stress and obsessive-compulsive disorder

The results also revealed a posiotive correlation between perceived stress and obsessive compulsive-disorder. Studies have indicated that the prevalence of perceived stress, anxiety, depression, and obsessive-compulsive symptoms increased significantly during the Covid-19 epidemic [47]. Kracker Imthon et al. [48] found that of the 1001 OCD patients, 605 (60.5%) reported experienceing at least one stressful life event during their lifetime. These results suggest that stressful life events may make Brazilian OCD patients more vulnerable to the onset

or exacerbation of obsessive-compulsive symptoms [48]. The results of a study conducted by ElliKoumantarou Malisiova on obsessive-compulsive patients also showed this correlation [49]. Obsessive Compulsive Disorder (OCD) may be the result of an interaction between genetic and environmental risk factors, including exposure to stressful and life-threatening events such as age-related changes in the reproductive systems, parenting styles, social and economic problems [50, 51]. Also, increased general stress (for example, job loss and family disease) and changes in normal life routines are among the causes of severe obsessive-compulsive disorder [48], and it is true about Covid-19 and the quarantine caused by it. The stress caused by the Covid-19 virus has caused many problems for people who are vulnerable to obsessive-compulsive disorder, as this behavior disrupts the normal course of their lives [52].

The correlation between obsessive-compulsive disorder and quality of life

The results showed a negative correlation between obsessive-compulsive disorder and quality of life. In previous epidemics such as Acute Respiratory Syndrome (SARS), Middle East Respiratory Syndrome (MERS), and the influenza, exacerbations of OCD have been reported 6 to 12 months after its outbreak. When coping strategies involved repetitive behaviors, there is a risk of increasing obsessive-compulsive disorder [52]. Shaoo et al. [53] found that patients with OCD scored significantly less in total score as well as all the domains of quality of life compared to healthy control group subjects. The main characteristic of obsessive-compulsive disorder (OCD) is the presence of recurrent or severe obsessions or compulsions that cause considerable suffering for person. These obsessions cause significant disruption in the normal process of life, normal social activities, and relationships [54] and thus negatively affect a person's quality of life. OCD as a disorder affects the quality of life in domains of mental health such as social functioning and role limitation due to emotional problems and mental health [55].

Study limitations

The first limitation of this study is that we selected participants to study through an online survey platform, those who do not have access to online surveys (e.g., the elderly), people who do not have access to the internet and people with low literacy, entered the study less than others and the findings may be prone to selection bias. Second, psychological health was reported by the participants themselves, and no acceptable evaluation tool was used. Third, the data in this study were self-reported, and participants' responses may be prone to social desirability bias. Therefore, future studies are suggested to design and implement intervention studies in the form of some models and theories such as social cognitive theory, protection motivation theory and stress management theory. Also, this study is recommended in other populations (such as patients recuperated from Covid-19 or individuals who have lost a family member due to Covid-19 disease) and with other psychological variables (such as depression, Post traumatic stress disorder (PTSD) or prolonged grief disorder).

Study strengths

-Data collection from 2 provinces of Iran (a city with commercial ports and an industrial city and a city with a lot of tourist attractions).

-Using the validated questionnaires

-Synchronization time coincides with pandemic peak time

Conclusion

The present findings show that the outbreak of coronavirus has changed people's living conditions and has had devastating psychological effects such as stress, health anxiety and obsessive behaviors, resulting in reduced quality of life. Thus, the design of psychological interventions to improve the mental health of individuals during the disease has a particular

importance and the necessary measures should be taken by policy makers and relevant officials in this regard. It is necessary to implement counseling and psychological interventions at the community level in the current situation, interventions such as: creating support networks with the presence of a counselor and a psychologist, telephone counseling, psychological support for medical staff, patients recuperated from Covid-19 or individuals who have lost a family member due to Covid-19 disease and so on.

Acknowledgements

The authors would like to thank all the participants in the study.

References

1. Bernard I, Limonta D, Mahal LK, Hobman TC. Endothelium infection and dysregulation by SARS-CoV-2: Evidence and caveats in COVID-19. Viruses. 2021;13(1):29.
2. Wu Z, McGoogan JM. Characteristics of and important lessons from the coronavirus disease 2019 (COVID-19) outbreak in China: summary of a report of 72 314 cases from the Chinese Center for Disease Control and Prevention. Jama. 2020;323(13):1239-42.
3. Moradzadeh R. The challenges and considerations of community-based preparedness at the onset of COVID-19 outbreak in Iran, 2020. Epidemiology & Infection. 2020;148.
4. WHO. WHO Coronavirus Disease (COVID-19) Dashboard Data 2021 .
5. Alizadehfard S, Alipuor A. The path analysis model in prediction of corona phobia based on intolerance of uncertainly and health anxiety. Journal of Research in Psychological Health. 2020;14(1):16-27.
6. Brooks SK, Webster RK, Smith LE, Woodland L, Wessely S, Greenberg N, et al. The psychological impact of quarantine and how to reduce it: rapid review of the evidence. The lancet. 2020;395(10227):912-20.

7. Wang Y, Xu B, Zhao G, Cao R, He X, Fu S. Is quarantine related to immediate negative psychological consequences during the 2009 H1N1 epidemic? General hospital psychiatry. 2011;33(1):75-7.

8. Rubin GJ, Wessely S. The psychological effects of quarantining a city. Bmj. 2020;368.

9. van Ballegooijen H, Goossens L, Bruin RH, Michels R, Krol M. Concerns, quality of life, access to care and productivity of the general population during the first 8 weeks of the coronavirus lockdown in Belgium and the Netherlands. BMC health services research. 2021;21(1):1-8.

10. Zhang W-r, Wang K, Yin L, Zhao W-f, Xue Q, Peng M, et al. Mental health and psychosocial problems of medical health workers during the COVID-19 epidemic in China. Psychotherapy and psychosomatics. 2020;89(4):242-50.

11. Shahyad S, Mohammadi MT. Psychological impacts of Covid-19 outbreak on mental health status of society individuals: a narrative review. Journal of Military Medicine. 2020;22(2):184-92.

12. Zarabadipour M, Asgari Ghonche MR, Asgari Ghonche S, Mirzadeh M. Psychological evaluation of the factors affecting the stress caused by COVID-19 outbreak in the medical staff and the community of Qazvin, Iran Spring 2020. Journal Mil Med. 2020;22(6):517-25.

13. Qi M, Li P, Moyle W, Weeks B, Jones C. Physical activity, health-related quality of life, and stress among the Chinese adult population during the COVID-19 pandemic. International journal of environmental research and public health. 2020;17(18):6494.

14. Asmundson GJ, Abramowitz JS, Richter AA, Whedon M. Health anxiety: current perspectives and future directions. Current psychiatry reports. 2010;12(4):306-12.

15. Kosic A, Lindholm P, Järvholm K, Hedman-Lagerlöf E, Axelsson E. Three decades of increase in health anxiety: Systematic review and meta-analysis of birth cohort changes in university student samples from 1985 to 2017. Journal of Anxiety Disorders. 2020;71:102208.

16. Asmundson GJ, Taylor S. How health anxiety influences responses to viral outbreaks like COVID-19: What all decision-makers, health authorities, and health care professionals need to know. Journal of anxiety disorders. 2020;71:102211.

17. Taylor S. The psychology of pandemics: Preparing for the next global outbreak of infectious disease: Cambridge Scholars Publishing; 2019.

18. M F. Psychological consequences of coronavirus outbreak in the community. Social Impact Assessment. 2020;1(2):207-25.

19. Abdelghani M, Mahdy R, El-Gohari H. Health anxiety to COVID-19 virus infection and its relationship to quality of life in a sample of health care workers in Egypt: a cross-sectional study. Archives of Psychiatry and Psychotherapy. 2021;23(1):19-28.

20. Seçer İ, Ulaş S. An investigation of the effect of COVID-19 on OCD in youth in the context of emotional reactivity, experiential avoidance, depression and anxiety. International journal of mental health and addiction. 2020:1-14.

21. Abramowitz JS, Jacoby RJ. Obsessive-compulsive disorder in the DSM-5. Clinical Psychology: Science and Practice. 2014;21(3):221.

22. Shafran R, Coughtrey A, Whittal M. Recognising and addressing the impact of COVID-19 on obsessive-compulsive disorder. The Lancet Psychiatry. 2020;7(7):570-2.

23. Abba-Aji A, Li, D., Hrabok, M., Shalaby, R., Gusnowski, A., Vuong, W., ... & Agyapong, V. I. COVID-19 pandemic and mental health: prevalence and correlates of new-onset obsessive-compulsive symptoms in a Canadian province. International Journal of Environmental Research and Public Health. 2020;17(19):6986.

24. Alimohammadi F, Setodeh-asl N, Karami A. Designing a model of quality of life in elderly based on perceived stress and tolerance of distress. Journal of Health and Care. 2019;21(1):53-65.

25. Salkovskis PM, Rimes KA, Warwick H, Clark D. The Health Anxiety Inventory: development and validation of scales for the measurement of health anxiety and hypochondriasis. Psychological medicine. 2002;32(5):843-53.

26. Abramowitz JS, Olatunji BO, Deacon BJ. Health anxiety, hypochondriasis, and the anxiety disorders. Behavior Therapy. 2007;38(1):86-94.

27. Cohen S, Kamarck T, Mermelstein R. A global measure of perceived stress. Journal of health and social behavior. 1983:385-96.

28. Taghavi SM, Mokarami H, Nazifi M, Choobineh A, Marioryad H, Kujerdi MF. The influence of socio-demographic, health and work-related factors on health-related quality of life among Iranian industrial workers. Health. 2014;2014.

29. Usefy A, Ghassemi GR, Sarrafzadegan N, Mallik S, Baghaei A, Rabiei K. Psychometric properties of the WHOQOL-BREF in an Iranian adult sample. Community mental health journal. 2010;46(2):139-47.

30. Sanavio E. Obsessions and compulsions: the Padua Inventory. Behaviour research and therapy. 1988;26(2):169-77.

31. Van Oppen P. Obsessions and compulsions: dimensional structure, reliability, convergent and divergent validity of the Padua Inventory. Behaviour Research and Therapy. 1992;30(6):631-7.

32. Hayter AL, Salkovskis PM, Silber E, Morris RG. The impact of health anxiety in patients with relapsing remitting multiple sclerosis: Misperception, misattribution and quality of life. British Journal of Clinical Psychology. 2016;55(4):371-86.

33. Trougakos JP, Chawla N, McCarthy JM. Working in a pandemic: Exploring the impact of COVID-19 health anxiety on work, family, and health outcomes. Journal of Applied Psychology. 2020.

34. Korkmaz S, Kazgan A, Çekiç S, Tartar AS, Balcı HN, Atmaca M. The anxiety levels, quality of sleep and life and problem-solving skills in healthcare workers employed in COVID-19 services. Journal of Clinical Neuroscience. 2020;80:131-6.

35. Shams JH, Mohammadzadeh KA, Maher A. Correlation between Health Literacy and quality of life with health anxiety in outpatient patients referred to Shahid Beheshti Specialized Polyclinic in Karaj. J Health Promot Manag. 2020;9(1):1-9.

36. Taylor S, Asmundson GJ, Hyprochondria. Treating health anxiety: A cognitive-behavioral approach: Guilford Press New York; 2004.

37. Sica C, Caudek C, Cerea S, Colpizzi I, Caruso M, Giulini P, et al. Health anxiety predicts the perceived dangerousness of covid-19 over and above intrusive illness-related thoughts, contamination symptoms, and state and trait negative affect. International Journal of Environmental Research and Public Health. 2021;18(4):1933.

38. Dennis D, Radnitz C, Wheaton MG. A Perfect Storm? Health Anxiety, Contamination Fears, and COVID-19: Lessons Learned from Past Pandemics and Current Challenges. International journal of cognitive therapy. 2021:1-17.

39. Piacentini J, Langley AK. Cognitive-behavioral therapy for children who have obsessive-compulsive disorder. Journal of clinical psychology. 2004;60(11):1181-94.

40. Çelmeçe N, Menekay M. The effect of stress, anxiety and burnout levels of healthcare professionals caring for COVID-19 patients on their quality of life. Frontiers in psychology. 2020;11:3329.

41. Tejoyuwono AAT, Nugraha RP, Fahdi FK. The Effect of Mental Health Status on the Quality of Life of Faculty of Medicine Students during the Pandemic Coronavirus Disease 2019 Period. Open Access Macedonian Journal of Medical Sciences. 2021;9(E):645-52.

42. Racic M, Todorovic R, Ivkovic N, Masic S, Joksimovic B, Kulic M. Self-perceived stress in relation to anxiety, depression and health-related quality of life among health

professions students: a cross-sectional study from Bosnia and Herzegovina. Slovenian Journal of Public Health. 2017;56(4):251.

43. Garbóczy S, Szemán-Nagy A, Ahmad MS, Harsányi S, Ocsenás D, Rekenyi V, et al. Health anxiety, perceived stress, and coping styles in the shadow of the COVID-19. BMC psychology. 2021;9(1):1-13.

44. Svensson SJ, Elntib S. Community cohesion during the first peak of the COVID-19 pandemic: A social antidote to health anxiety and stress. Journal of Applied Social Psychology. 2021.

45. Wheaton MG, Abramowitz JS, Berman NC, Fabricant LE, Olatunji BO. Psychological predictors of anxiety in response to the H1N1 (swine flu) pandemic. Cognitive Therapy and Research. 2012;36(3):210-8.

46. Office for National Statistics. Coronavirus and the social impacts on Great Britain. 2020.

47. Mrklas K, Shalaby R, Hrabok M, Gusnowski A, Vuong W, Surood S, et al. Prevalence of perceived stress, anxiety, depression, and obsessive-compulsive symptoms in health care workers and other workers in Alberta during the COVID-19 pandemic: cross-sectional survey. JMIR Mental Health. 2020;7(9):e22408.

48. Kracker Imthon A, Antônio Caldart C, do Rosário MC, Fontenelle LF, Constantino Miguel E, Arzeno Ferrão Y. Stressful life events and the clinical expression of Obsessive–Compulsive Disorder (OCD): an exploratory study. Journal of clinical medicine. 2020;9(10):3371.

49. Malisiova EK, Mourikis I, Chalimourdas T, Nianiakas N, Michou M, Mantzou A, et al. Low hair cortisol concentrations in obsessive compulsive disorder: a cross-sectional study. Journal of Psychiatric Research. 2020;131:187-93.

50. Adams TG, Kelmendi B, Brake CA, Gruner P, Badour CL, Pittenger C. The role of stress in the pathogenesis and maintenance of obsessive-compulsive disorder. Chronic Stress. 2018;2:2470547018758043.

51. Brander G, Perez-Vigil A, Larsson H, Mataix-Cols D. Systematic review of environmental risk factors for obsessive-compulsive disorder: a proposed roadmap from association to causation. Neuroscience & Biobehavioral Reviews. 2016;65:36-62.

52. Banerjee D. The other side of COVID-19: Impact on obsessive compulsive disorder (OCD) and hoarding. Psychiatry research. 2020;288:112966.

53. Sahoo P, Sethy RR, Ram D. Functional impairment and quality of life in patients with obsessive compulsive disorder. Indian journal of psychological medicine. 2017;39(6):760-5.

54. Kaplan HI, Sadock BJ. Synopsis of psychiatry: Behavioral sciences clinical psychiatry: Williams & Wilkins Co; 1988.

55. Koran LM, Thienemann ML, Davenport R. Quality of life for patients with obsessive-compulsive disorder. The American journal of psychiatry. 1996.

Dear Editor in Chief,
Journal of PLOS ONE
Thank you for giving us this opportunity to revise the manuscript entitled: "Quality of life and its related psychological problems during coronavirus pandemic". The corrections have been made in the manuscript based on Comments.

We used the yellow color to highlight the requested revisions by the honorable reviewer 1, the bright green to highlight the requested revisions by the honorable reviewer 2, the dark yellow color to highlight the requested revisions by the honorable reviewer 3, and the turquoise color to highlight the common requested revisions by all of the honorable reviewers. We also used the pink color to highlight the requested revisions (journal requirements).

I hope they would be satisfactory.
I wish all the best for you
Shirin Hosseini, Ph.D
Corresponding Author

Reviewer Comments:
Honorable reviewer 1:
1.Please highlight your contribution and novelty of this manuscript with accuracy in the introduction part before the arrangement description.

- Our thanks to the honorable reviewer for your attention. Some explanations about the importance of this study and its novelty were presented in the last paragraph in the Introduction section.
- Regarding the importance and novelty of this study and the necessity of doing it, the following can be mentioned:

1. Coronavirus disease 2019 is a new subject and its effects are diverse in different people. This study has examined various psychological variables and their relationships with quality of life during coronavirus pandemic (especially health anxiety and its relationship with obsessive-compulsive disorder) which has been less studied in other studies. This study also has examined stress and health anxiety separately and few studies have addressed these two types of emotional problems separately.
2. This study concerns a population very affected by the Covid-19 pandemic. Vaccination against covid-19 started later in Iran than in other countries in the world. Delay in starting vaccination, people's negligence in observing health protocols, their resistance to vaccination and other reasons, led to higher prevalence and mortality due to the coronavirus in Iran, and as a result, imposed extra psychological pressure on people.
3. This research was conducted in two provinces of Hormozgan and Isfahan. These cities were more vulnerable to coronavirus, for some reasons: Isfahan is an industrial city and a city with a lot of tourist attractions and Hormozgan is a city with commercial ports. The hot weather, the impossibility of proper ventilation especially during the summer, and social gathering in closed environments led to the prevalence of covid-19 in Hormozgan. Besides, high humidity made it difficult for people to use face masks outdoors. In addition, in Iran, the cultural background of people, who are known to hospitable, was also influential in the increased prevalence of this disease. Generally, hospitality is more prominent in southern provinces of Iran, especially in Hormozgan province. All of this contributes to the high prevalence of covid-19 and further psychological problems in these cities.

2. The literature and theoretical background should improve and add more relevant studies e.g. (latest) to grab and display more contemporary literature critically.

- Our thanks to the honorable reviewer for your attention. According to the honorable reviewer's suggestion, the literature and theoretical background was improved and more relevant studies were added.

3. Please check your manuscript for errors. On first glance at your manuscript, we noticed several grammar errors. Please check the whole paper carefully or have it professionally edited.

- Our thanks to the honorable reviewer for your attention. According to the honorable reviewer's suggestion, the manuscript was professionally edited.

4. In the discussion part, you must contact the results, and some of the content needs to be rewritten.

- Our thanks to the honorable reviewer for your attention. Some changes made in the discussion section. We added sub-headings to the Discussion section to guide the readers through this section. We wrote the discussion section as follows: we summarized the important findings in the first paragraph. We presented the findings separately in several paragraphs and compared them with the results of previous studies. And finally we explained the obtained findings and explained why such a result was achieved.

5. It is suggested to use advanced statistical methods to add the influencing factors on the psychological changes of the population. For example, the potential influencing factors were analyzed by logistic regression.

- Our thanks to the honorable reviewer for your attention. We used path analysis and structural equation modeling (SEM) to test the relationships between the variables. Regarding the suggestion of honorable reviewer (using logistic regression), it should be noted that logistic regression is used to predict a multi-category dependent variable. However, "quality of life" as a dependent variable in this study, is not a multi-category variable. Based on the explanations provided, does the honorable reviewer suggest a specific statistical test?

6. Note the order of Table 3 and table 4 in the article.

- Our thanks to the honorable reviewer for your attention. The order of table 3 and table 4 in the article was modified.

Honorable Reviewer 2:

1.This study entitled "Quality of life and its related psychological problems during coronavirus pandemic" aimed to assess that relationship in a population of adult people in Iran. This topic has been extensively covered in the literature over the past two years. The correlation between quality of life and mental health has been extensively studied to date. Although this study concerns a population very affected by the covid-19 pandemic, the sample studied does not seem to fully correspond to the population from which it is extracted.

- Our thanks to the honorable reviewer for your attention. Regarding the importance and novelty of this study and the necessity of doing it, the following can be mentioned:

4. Coronavirus disease 2019 is a new subject and its effects are diverse in different people. This study has examined various psychological variables and their relationships with quality of life during coronavirus pandemic (especially health anxiety and its relationship with obsessive-compulsive disorder) which has been less studied in other studies. This study also has examined stress and health anxiety separately and few studies have addressed these two types of emotional problems separately.

5. As the honorable reviewer pointed out, this study concerns a population very affected by the Covid-19 pandemic. Vaccination against covid-19 started later in Iran than in other countries in the world. Delay in starting vaccination, people's negligence in observing health protocols, their resistance to vaccination, economic problems caused by the coronavirus and other reasons, led to higher prevalence and mortality due to the coronavirus in Iran, and as a result, imposed extra psychological pressure on people.
6. This research was conducted in two provinces of Hormozgan and Isfahan. These cities were more vulnerable to coronavirus, for some reasons: Isfahan is an industrial city and a city with a lot of tourist attractions and Hormozgan is a city with commercial ports. The hot weather, the impossibility of proper ventilation especially during the summer, and social gathering in closed environments led to the prevalence of covid-19 in Hormozgan. Besides, high humidity made it difficult for people to use face masks outdoors. In addition, in Iran, the cultural background of people, who are known to hospitable, was also influential in the increased prevalence of this disease. Generally, hospitality is more prominent in southern provinces of Iran, especially in Hormozgan province. All of this contributes to the high prevalence of covid-19 and further psychological problems in these cities.

Some explanations about the importance of this study and its novelty were presented in the last paragraph in the Introduction section.

2. In addition, the demographic characteristics collected are not sufficient to give a complete view of the sample.

- Our thanks to the honorable reviewer for your attention. The most important demographic characteristics related to the purpose of our study (and based on similar studies) were collected. Does the honorable reviewer have a special demographic characteristic in mind?

3.A big lack of data concerns the period in which the study was conducted, or rather in which the responses from the participants were collected.

- Our thanks to the honorable reviewer for your attention. The data collection was conducted from October 22, 2020 to February 3, 2021.

4.In addition, the convenience sample and the method of recruiting participants represent possible selection biases (the recruitment method has not been clearly specified).

- Our thanks to the honorable reviewer for your attention. Further details on data collection are provided in Materials and Methods (Sample size and sampling procedure section). It should be noted that due to the prevalence of coronavirus and it severity during the study (data collection) in Iran and the need to compliance with quarantine, we had to collect data through social media. Otherwise, both the researchers and the participants would have to pay heavy fines. In study limitations, we have mentioned that one of the limitations of this study is that the questionnaire survey was conducted only via the internet, which was only available to participants who were literate and had access to the internet.

5.Language must be deeply revised.

- Our thanks to the honorable reviewer for your attention. According to the honorable reviewer's suggestion, language was deeply revised.

6.The Discussion section lack of "linearity"; it is unclear, with statements followed by some bibliographical references, without a logic and concise semantic structure.

- Our thanks to the honorable reviewer for your attention. Some changes made in the discussion section. We added sub-headings to the Discussion section to guide the readers through this section. We wrote the discussion section as follows: we summarized the important findings in the first paragraph. We presented the findings separately in several paragraphs and compared them with the results of previous studies. And finally we explained the obtained findings and explained why such a result was achieved.

7. The statistical results are also presented in an unclear way.

- Our thanks to the honorable reviewer for your attention. Some explanations about the results presented in each table, are provided at the bottom of the table.

8. The declared limitations are quite slight, just as the few strengths of the study were not highlighted (for example the use of validated questionnaires).

- Our thanks to the honorable reviewer for your attention. We highlighted the study strengths and modified the study limitations.

9. The conclusion, finally, lack of strength and "interest".

- Our thanks to the honorable reviewer for your attention. We made some changes in the conclusion section.

Honorable Reviewer 3:
Thank you for giving me the opportunity to review this article. Here are some questions to the authors. I think this manuscript has an appropriate introduction and well justified.

1.There are some repetitive data and statements in the abstract (result) that is better to modify.
Our thanks to the honorable reviewer for your attention. According to the honorable reviewer's suggestion, the repetitive data and statements in the Abstract (result section) was modified.

2. Participants: How authors are certain the sample population have no OCOD? I know it is mentioned in limitations but self-disclosure is not enough.

- Our thanks to the honorable reviewer for your attention. It should be noted that individuals who had no history of taking any psychiatric medication were included in the study. In other words, the authors examined this criterion (not suffering from psychological disorders such as obsessive-compulsive disorder) by asking this question: **"Do you have a history of taking psychiatric medications?".**

3. I suggest that The importance of this study and its novelty should be explain more.

- Our thanks to the honorable reviewer for your attention. Some explanations about the importance of this study and its novelty were presented in the last paragraph in the Introduction section.
- Regarding the importance and novelty of this study and the necessity of doing it, the following can be mentioned:

1.Coronavirus disease 2019 is a new subject and its effects are diverse in different people. This study has examined various psychological variables and their relationships with quality of life during coronavirus pandemic (especially health anxiety and its relationship with obsessive-

compulsive disorder) which has been less studied in other studies. This study also has examined stress and health anxiety separately and few studies have addressed these two types of emotional problems separately.
2.This study concerns a population very affected by the Covid-19 pandemic. Vaccination against covid-19 started later in Iran than in other countries in the world. Delay in starting vaccination, people's negligence in observing health protocols, their resistance to vaccination and other reasons, led to higher prevalence and mortality due to the coronavirus in Iran, and as a result, imposed extra psychological pressure on people.
3.This research was conducted in two provinces of Hormozgan and Isfahan. These cities were more vulnerable to coronavirus, for some reasons: Isfahan is an industrial city and a city with a lot of tourist attractions and Hormozgan is a city with commercial ports. The hot weather, the impossibility of proper ventilation especially during the summer, and social gathering in closed environments led to the prevalence of covid-19 in Hormozgan. Besides, high humidity made it difficult for people to use face masks outdoors. In addition, in Iran, the cultural background of people, who are known to hospitable, was also influential in the increased prevalence of this disease. Generally, hospitality is more prominent in southern provinces of Iran, especially in Hormozgan province. All of this contributes to the high prevalence of covid-19 and further psychological problems in these cities.

4. In my opinion statistical analyses are very complete and all well detailed.

- Our thanks to the honorable reviewer for your attention.

5. In the discussion perhaps it is missing to indicate future lines of research.

- Our thanks to the honorable reviewer for your attention. Some suggestions for future studies were presented in the discussion section, at the end of "study limitations" part.
