# Supplementary material for: Quality of life and its related psychological problems during coronavirus pandemic
Source: PLoS One. 2022 Oct 31;17(10):e0276841. doi: 10.1371/journal.pone.0276841 (PMC9621443; doi:10.1371/journal.pone.0276841)
Supplement: Attachment — Submitted filename: 1.docx [file pone.0276841.s002.docx]

Dear editor

I have carefully considered and read the manuscript entitled " Quality of life and its related psychological problems during coronavirus pandemic

" and have the following observations:

The article focuses on the impact of the epidemic situation on the psychological state of the population, which is an interesting point. However, the manuscript is a descriptive study, which is limited. It is suggested to add the limitations of the article in the discussion.

1. Please highlight your contribution and novelty of this manuscript with accuracy in the introduction part before the arrangement description.

2. The literature and theoretical background and should improve and add more relevant studies e.g. (latest) to grab and display more contemporary literature critically.

3. Please check your manuscript for errors. On first glance at your manuscript, we noticed several grammar errors. Please check the whole paper carefully or have it professionally edited.

4. In the discussion part, you must contact the results, and some of the content needs to be rewritten.

5. It is suggested to use advanced statistical methods to add the influencing factors on the psychological changes of the population. For example, the potential influencing factors were analyzed by logitic regression

6. Note the order of Table 3 and table 4 in the article
