# Supplementary material for: Quality of life and its related psychological problems during coronavirus pandemic
Source: PLoS One. 2022 Oct 31;17(10):e0276841. doi: 10.1371/journal.pone.0276841 (PMC9621443; doi:10.1371/journal.pone.0276841)
Supplement: Attachment — Submitted filename: response to reviewers.docx [file pone.0276841.s003.docx]

Dear Editor in Chief,

Journal of PLOS ONE

Thank you for giving us this opportunity to revise the manuscript entitled: "Quality of life and its related psychological problems during coronavirus pandemic". The corrections have been made in the manuscript based on Comments.

We used the yellow color to highlight the requested revisions by the honorable reviewer 1, the bright green to highlight the requested revisions by the honorable reviewer 2, the dark yellow color to highlight the requested revisions by the honorable reviewer 3, and the turquoise color to highlight the common requested revisions by all of the honorable reviewers. We also used the pink color to highlight the requested revisions (journal requirements).

I hope they would be satisfactory.

I wish all the best for you

Shirin Hosseini, Ph.D

Corresponding Author

**Reviewer Comments:**

Honorable reviewer 1:

1.Please highlight your contribution and novelty of this manuscript with accuracy in the introduction part before the arrangement description.

- Our thanks to the honorable reviewer for your attention. Some explanations about the importance of this study and its novelty were presented in the last paragraph in the Introduction section.
- Regarding the importance and novelty of this study and the necessity of doing it, the following can be mentioned:

1. Coronavirus disease 2019 is a new subject and its effects are diverse in different people. This study has examined various psychological variables and their relationships with quality of life during coronavirus pandemic (especially health anxiety and its relationship with obsessive-compulsive disorder) which has been less studied in other studies. This study also has examined stress and health anxiety separately and few studies have addressed these two types of emotional problems separately.
2. This study concerns a population very affected by the Covid-19 pandemic. Vaccination against covid-19 started later in Iran than in other countries in the world. Delay in starting vaccination, people's negligence in observing health protocols, their resistance to vaccination and other reasons, led to higher prevalence and mortality due to the coronavirus in Iran, and as a result, imposed extra psychological pressure on people.
3. This research was conducted in two provinces of Hormozgan and Isfahan. These cities were more vulnerable to coronavirus, for some reasons: Isfahan is an industrial city and a city with a lot of tourist attractions and Hormozgan is a city with commercial ports. The hot weather, the impossibility of proper ventilation especially during the summer, and social gathering in closed environments led to the prevalence of covid-19 in Hormozgan. Besides, high humidity made it difficult for people to use face masks outdoors. In addition, in Iran, the cultural background of people, who are known to hospitable, was also influential in the increased prevalence of this disease. Generally, hospitality is more prominent in southern provinces of Iran, especially in Hormozgan province. All of this contributes to the high prevalence of covid-19 and further psychological problems in these cities.

2. The literature and theoretical background should improve and add more relevant studies e.g. (latest) to grab and display more contemporary literature critically.

- Our thanks to the honorable reviewer for your attention. According to the honorable reviewer's suggestion, the literature and theoretical background was improved and more relevant studies were added.

3. Please check your manuscript for errors. On first glance at your manuscript, we noticed several grammar errors. Please check the whole paper carefully or have it professionally edited.

- Our thanks to the honorable reviewer for your attention. According to the honorable reviewer's suggestion, the manuscript was professionally edited.

4. In the discussion part, you must contact the results, and some of the content needs to be rewritten.

- Our thanks to the honorable reviewer for your attention. Some changes made in the discussion section. We added sub-headings to the Discussion section to guide the readers through this section. We wrote the discussion section as follows: we summarized the important findings in the first paragraph. We presented the findings separately in several paragraphs and compared them with the results of previous studies. And finally we explained the obtained findings and explained why such a result was achieved.

5. It is suggested to use advanced statistical methods to add the influencing factors on the psychological changes of the population. For example, the potential influencing factors were analyzed by logistic regression.

- Our thanks to the honorable reviewer for your attention. We used path analysis and structural equation modeling (SEM) to test the relationships between the variables. Regarding the suggestion of honorable reviewer (using logistic regression), it should be noted that logistic regression is used to predict a multi-category dependent variable. However, "quality of life" as a dependent variable in this study, is not a multi-category variable. Based on the explanations provided, does the honorable reviewer suggest a specific statistical test?

6. Note the order of Table 3 and table 4 in the article.

- Our thanks to the honorable reviewer for your attention. The order of table 3 and table 4 in the article was modified.

Honorable Reviewer 2:

1.This study entitled "Quality of life and its related psychological problems during coronavirus pandemic" aimed to assess that relationship in a population of adult people in Iran. This topic has been extensively covered in the literature over the past two years. The correlation between quality of life and mental health has been extensively studied to date. Although this study concerns a population very affected by the covid-19 pandemic, the sample studied does not seem to fully correspond to the population from which it is extracted.

- Our thanks to the honorable reviewer for your attention. Regarding the importance and novelty of this study and the necessity of doing it, the following can be mentioned:

4. Coronavirus disease 2019 is a new subject and its effects are diverse in different people. This study has examined various psychological variables and their relationships with quality of life during coronavirus pandemic (especially health anxiety and its relationship with obsessive-compulsive disorder) which has been less studied in other studies. This study also has examined stress and health anxiety separately and few studies have addressed these two types of emotional problems separately.
5. As the honorable reviewer pointed out, this study concerns a population very affected by the Covid-19 pandemic. Vaccination against covid-19 started later in Iran than in other countries in the world. Delay in starting vaccination, people's negligence in observing health protocols, their resistance to vaccination, economic problems caused by the coronavirus and other reasons, led to higher prevalence and mortality due to the coronavirus in Iran, and as a result, imposed extra psychological pressure on people.
6. This research was conducted in two provinces of Hormozgan and Isfahan. These cities were more vulnerable to coronavirus, for some reasons: Isfahan is an industrial city and a city with a lot of tourist attractions and Hormozgan is a city with commercial ports. The hot weather, the impossibility of proper ventilation especially during the summer, and social gathering in closed environments led to the prevalence of covid-19 in Hormozgan. Besides, high humidity made it difficult for people to use face masks outdoors. In addition, in Iran, the cultural background of people, who are known to hospitable, was also influential in the increased prevalence of this disease. Generally, hospitality is more prominent in southern provinces of Iran, especially in Hormozgan province. All of this contributes to the high prevalence of covid-19 and further psychological problems in these cities.

Some explanations about the importance of this study and its novelty were presented in the last paragraph in the Introduction section.

2. In addition, the demographic characteristics collected are not sufficient to give a complete view of the sample.

- Our thanks to the honorable reviewer for your attention. The most important demographic characteristics related to the purpose of our study (and based on similar studies) were collected. Does the honorable reviewer have a special demographic characteristic in mind?

3.A big lack of data concerns the period in which the study was conducted, or rather in which the responses from the participants were collected.

- Our thanks to the honorable reviewer for your attention. The data collection was conducted from October 22, 2020 to February 3, 2021.

4.In addition, the convenience sample and the method of recruiting participants represent possible selection biases (the recruitment method has not been clearly specified).

- Our thanks to the honorable reviewer for your attention. Further details on data collection are provided in Materials and Methods (Sample size and sampling procedure section). It should be noted that due to the prevalence of coronavirus and it severity during the study (data collection) in Iran and the need to compliance with quarantine, we had to collect data through social media. Otherwise, both the researchers and the participants would have to pay heavy fines. In study limitations, we have mentioned that one of the limitations of this study is that the questionnaire survey was conducted only via the internet, which was only available to participants who were literate and had access to the internet.

5.Language must be deeply revised.

- Our thanks to the honorable reviewer for your attention. According to the honorable reviewer's suggestion, language was deeply revised.

6.The Discussion section lack of "linearity"; it is unclear, with statements followed by some bibliographical references, without a logic and concise semantic structure.

- Our thanks to the honorable reviewer for your attention. Some changes made in the discussion section. We added sub-headings to the Discussion section to guide the readers through this section. We wrote the discussion section as follows: we summarized the important findings in the first paragraph. We presented the findings separately in several paragraphs and compared them with the results of previous studies. And finally we explained the obtained findings and explained why such a result was achieved.

7. The statistical results are also presented in an unclear way.

- Our thanks to the honorable reviewer for your attention. Some explanations about the results presented in each table, are provided at the bottom of the table.

8. The declared limitations are quite slight, just as the few strengths of the study were not highlighted (for example the use of validated questionnaires).

- Our thanks to the honorable reviewer for your attention. We highlighted the study strengths and modified the study limitations.

9. The conclusion, finally, lack of strength and "interest".

- Our thanks to the honorable reviewer for your attention. We made some changes in the conclusion section.

Honorable Reviewer 3:

Thank you for giving me the opportunity to review this article. Here are some questions to the authors. I think this manuscript has an appropriate introduction and well justified.

1.There are some repetitive data and statements in the abstract (result) that is better to modify.
Our thanks to the honorable reviewer for your attention. According to the honorable reviewer's suggestion, the repetitive data and statements in the Abstract (result section) was modified.

2. Participants: How authors are certain the sample population have no OCOD? I know it is mentioned in limitations but self-disclosure is not enough.

- Our thanks to the honorable reviewer for your attention. It should be noted that individuals who had no history of taking any psychiatric medication were included in the study. In other words, the authors examined this criterion (not suffering from psychological disorders such as obsessive-compulsive disorder) by asking this question: **"Do you have a history of taking psychiatric medications?".**

3. I suggest that The importance of this study and its novelty should be explain more.

- Our thanks to the honorable reviewer for your attention. Some explanations about the importance of this study and its novelty were presented in the last paragraph in the Introduction section.
- Regarding the importance and novelty of this study and the necessity of doing it, the following can be mentioned:

1.Coronavirus disease 2019 is a new subject and its effects are diverse in different people. This study has examined various psychological variables and their relationships with quality of life during coronavirus pandemic (especially health anxiety and its relationship with obsessive-compulsive disorder) which has been less studied in other studies. This study also has examined stress and health anxiety separately and few studies have addressed these two types of emotional problems separately.

2.This study concerns a population very affected by the Covid-19 pandemic. Vaccination against covid-19 started later in Iran than in other countries in the world. Delay in starting vaccination, people's negligence in observing health protocols, their resistance to vaccination and other reasons, led to higher prevalence and mortality due to the coronavirus in Iran, and as a result, imposed extra psychological pressure on people.

3.This research was conducted in two provinces of Hormozgan and Isfahan. These cities were more vulnerable to coronavirus, for some reasons: Isfahan is an industrial city and a city with a lot of tourist attractions and Hormozgan is a city with commercial ports. The hot weather, the impossibility of proper ventilation especially during the summer, and social gathering in closed environments led to the prevalence of covid-19 in Hormozgan. Besides, high humidity made it difficult for people to use face masks outdoors. In addition, in Iran, the cultural background of people, who are known to hospitable, was also influential in the increased prevalence of this disease. Generally, hospitality is more prominent in southern provinces of Iran, especially in Hormozgan province. All of this contributes to the high prevalence of covid-19 and further psychological problems in these cities.

4. In my opinion statistical analyses are very complete and all well detailed.

- Our thanks to the honorable reviewer for your attention.

5. In the discussion perhaps it is missing to indicate future lines of research.

- Our thanks to the honorable reviewer for your attention. Some suggestions for future studies were presented in the discussion section, at the end of "study limitations" part.
