# Supplementary material for: Quality of life and its related psychological problems during coronavirus pandemic
Source: PLoS One. 2022 Oct 31;17(10):e0276841. doi: 10.1371/journal.pone.0276841 (PMC9621443; doi:10.1371/journal.pone.0276841)
Supplement: Attachment — Submitted filename: Response to Reviewers.docx [file pone.0276841.s005.docx]

Dear Editor in Chief,

Journal of PLOS ONE

Thank you for giving us this opportunity to revise the manuscript entitled: "Quality of life and its related psychological problems during coronavirus pandemic". The corrections have been made in the manuscript based on Comments.

We used the yellow color to highlight the requested revisions by the honorable reviewer 4, the bright green to highlight the requested revisions by the honorable reviewer 5, the pink color to highlight the requested revisions by the honorable reviewer 6, and the turquoise color to highlight the common requested revisions by all of the honorable reviewers.

It should be noted that in the first revision, the honorable reviewer 2 asked us to add the strengths of the study to the conclusion. According to the honorable reviewer's suggestion, we added the "study strengths". But in the latest revision, the honorable reviewer 4 commented that "*there is no study strengths section in scientific papers and this section should be removed*". Meanwhile, two other honorable reviewers have emphasized the study strengths. According to these honorable reviewers' suggestion, we added the "study strengths" following the "study limitations" in the recent revision. We request help from the honorable editor regarding the removal or addition of the "study strengths."

I hope they would be satisfactory.

I wish all the best for you

Shirin Hosseini, Ph.D

Corresponding Author

**Reviewer #4: Dear Authors,**

I am pleased to study and review your study. The English proficiency of the manuscript is not appropriate, it should be re-written. I addressed many of them for your consideration. The statistical analysis is conducted incorrectly, you need to re-run the analyses as suggested and then write the results and discussion from scratch.
Good luck with the revision.

- Our thanks to the honorable reviewer for your complimentary comments and suggestions. According to the honorable reviewer's suggestions, all of the suggestions were applied in the manuscript and highlighted with yellow color. Language was deeply revised and the statistical analysis was run again as suggested.
- Regarding this comment of the honorable reviewer *"You clearly mentioned that you have measured the correlation between quality of life and obsessive-compulsive disorder, then it should be an inclusion criteria!"* at page 17, it should be noted that our purpose of considering this inclusion criterion was to exclude people from the study who were suffering from psychological disorders (such as OCD) even before the spread of Coronavirus. We wanted to investigate the prevalence of psychological disorders after the spread of Coronavirus and evaluate its impact on people's quality of life. So one of the inclusion criteria in our study was "not suffering from psychological disorders such as obsessive-compulsive disorder". We assess this criterion by a self-report question at the beginning of the questionnaire. Since we did not have access to the medical records of the participants, we relied on their self-reports (people who suffer from psychological disorders are mostly aware of their illness, and we assumed that these people are honest in answering this question regarding having psychological disorders). Individuals who confirmed that they do not have psychological disorders, their answers to the questions were examined and analyzed.
- Regarding this comment of the honorable reviewer "*These could not be considered as study strengths. The whole section should be removed, there is no "study strengths" section in scientific papers. If you want to emphasis on the importance of your study you need to add it to introduction section.",* it should be noted that in the first revision, the honorable reviewer 2 asked us to add the strengths of the study to the conclusion and in this revision, two other honorable reviewers have emphasized the study strengths. According to these honorable reviewers' suggestion, we added the "study strengths" following the "study limitations" in the recent revision. According to what has been said, what is the order of the honorable reviewer in this regard?

**Reviewer #5:** The study is an epidemiological cross-sectional exploratory study, looking into COVID-19-related anxiety, obsessions, and their link with the general population’s perceived quality of life. The sample size and characteristics make the results adequately generalizable. Although the study design, procedures, and findings do not show any novel features, the research question (if stated clearly) is well worth investigating among different populations and nationalities.

There are some minor concerns and comments which could improve the manuscript:

1-Keywords are rather irrelevant to the main aims of the study and are either too general or too specific.

- Our thanks to the honorable reviewer for your attention. All keywords were set based on MeSH. Does the honorable reviewer suggest special keywords?

2- Some typos across the manuscript – for instance, line 148, and 405.

- Our thanks to the honorable reviewer for your attention. These typos were corrected.

3- The introduction is sufficiently elaborative. However, the aims and hypotheses of this study are missing. I understand that it was a rather exploratory study, but adding the pre-assumptions and research questions could add a lot of value to your introduction. Why is this study conducted?

- Our thanks to the honorable reviewer for your attention. According to the honorable reviewer's suggestion, the specific research question and hypotheses of this study were written at the end of the Introduction section.

4- A self-report measure might not be the best way to exclude the participants with psychological disorders (e.g., OCD) ¬– it would have been better if you used a screening tool to exclude participants with psychiatric symptoms of OCD.

- Our thanks to the honorable reviewer for your attention. Since we did not have access to the medical records of the participants, we relied on their self-reports (people who suffer from psychological disorders are mostly aware of their illness, and we assumed that these people are honest in answering this question regarding having psychological disorders). We also used Padua inventory to assess OCD.

5-You used a snowball sampling method (as stated in line 177) – could you also report what standards you had for the snowballing? Were the participants aware of the inclusion/exclusion criteria for choosing whom to share the questionnaire with?

- Our thanks to the honorable reviewer for your attention. It should be noted that we used the cluster sampling method to collect data. This sentence was translated incorrectly. The residents of Isfahan participating in the study were 2 times more than the participants of Bandar Abbas.

6- What do you mean by the ‘underlying diseases’ in line 195, and how did you measure them?

- Our thanks to the honorable reviewer for your attention. The "underlying diseases" mean "chronic diseases" such as hypertension, diabetes, cardiovascular disease, kidney disease and etc. Because people who are suffering from chronic diseases are more exposed to COVID-19 and its complications than healthy people. We measured this item by a self-report question.

7- Table 3 is an unnecessarily exhaustive way of showing the correlation coefficients. You could perhaps summarize this table or convert it to a figure – namely a heat map.

- Our thanks to the honorable reviewer for your attention. We calculated the correlation coefficients only for main variables, and subscales of quality of life were removed.

8- Figure 1 has a low resolution and quality. Could it be improved resolution and graphical-wise? It could also benefit from a more informative caption.

- Our thanks to the honorable reviewer for your attention. We changed the caption of the figure. Does the honorable reviewer suggest a special and better caption?

9- I recommend separating the results section into subsections making it easier to follow. (could be classified as demographics, correlations, standardized estimations, etc.).

- Our thanks to the honorable reviewer for your attention. According to the honorable reviewer's suggestion, we separated the results section into subsections.

10- There are some inconsistencies across the manuscript regarding the name of the virus (coronavirus, COVID, Covid-19) – try to use a consistent term across the entire text.

- Our thanks to the honorable reviewer for your attention. According to the honorable reviewer's suggestion, we used a consistent term (COVID-19) for the name of the virus across the entire text.

11- I recommend reformatting the study strengths section to a text form rather than bullet-pointed items with possibly more elaborations (like why collecting data from a city with tourist attractions is considered a strength of this study?).

- Our thanks to the honorable reviewer for your attention. According to the honorable reviewer's suggestion, we reformatted the study strengths section to a text form with more elaborations. This section is followed by the "Study limitations" section. Regarding this study strength "collecting data from 2 provinces of Iran", it should be noted that these cities were more vulnerable to coronavirus, for some reasons: Isfahan is an industrial city and a city with a lot of tourism attractions and Bandar Abbas is a city with commercial ports. Due to its commercial ports, Banda Abbas has many immigrants from other provinces. The hot weather, the impossibility of proper ventilation especially during the summer, and social gathering in closed environments led to the prevalence of covid-19 in Bandar Abbas. Besides, high humidity made it difficult for people to use face masks outdoors. In addition, in Iran, the cultural background of people (who are known to hospitable), placing a lot of tourist attractions in Isfahan and the beautiful beaches in Bandar Abbas, has attracted more tourists and passengers to these cities, which is influential in the increased prevalence of this disease. All of this contributes to the high prevalence of covid-19 and further psychological problems in these cities. (Due to the prolongation of "limitations" section, we have presented these elaborations there. At the discretion of the honorable reviewer, these elaborations will be added to the manuscript.)

Overall, the study is an interesting one in light of the global complexities resulting from the pandemic and its influences on people's well-being. The manuscript could also benefit from a native English speaker for proofreading and revising some parts language-wise.

- Our thanks to the honorable reviewer for your complimentary comments and suggestions. According to the honorable reviewer's suggestion, language was deeply revised.

**Reviewer #6:** The research itself is well designed to test the interrelationships among the different variables of psychological distress during the Covid 19 pandemic in the Iranian population. The data collection tools and studied population were selected appropriately. However, a few points in the paper require further elaboration. This paper can be published after incorporating a few elements outlined below:

In Abstract: Results: please provide the value of r in decimals and mention the exact p-value. For example: (r = - or + 0. xx, p = 0.xxx) rather than just saying significant positive or negative correlations. The exact p-value is mandatory (look at the APA manual on reporting of r values). For reference, you can find similar information in this paper - <https://www.sciencedirect.com/science/article/pii/S2452247318302164>

- Our thanks to the honorable reviewer for your attention. According to the honorable reviewer's suggestion, we mentioned the exact p-value.

In the main text:1. The use of the language of the tools (short health anxiety inventory (SHAI), perceived stress scale (PSS), world health 52 organization quality of life questionnaire (WHOQOL-BREF) and Padua inventory)
- What was the language used for data collection? Persian/Farsi or English?
In the strength of the research, the authors have claimed that they have used validated tools. Were those tools validated in English or the local language? I believe that not all citizens of Iran could communicate well in the English language, if this is the case, the use of tools in the local language needs to have proper validation of the tools. The validation of tools in the local language is a long procedure and demands rigorous processes. In many cases, the authors have mentioned several studies have explored this and that, but no concrete findings or studies with explicit information on the language used, the validation process was done etc. were covered.

- Our thanks to the honorable reviewer for your attention. We used Persian language for data collection and all of the tools were validated in Persian (Shams et al, 2010; Karimi et al, 2015; Usefy et al, 2010; Maroufizadeh et al, 2014). More explanations about each questionnaire were presented in the "Research tools" section.

2. The collection of data from minors (below the age of 18) without the consent of their parents is a topic researcher should be aware of and avoid in any behavioural research. The information on the total number of children surveyed is not disclosed. It would be nice to clarify this topic.

- Our thanks to the honorable reviewer for your attention. We obtained consent from the participants themselves. Because in Iran, the population aged 15 and over is considered as an adult population. Therefore, according to the instructions of the Ethics Committee of the Hormozgan University of Medical Sciences, for participants aged 16-18, this consent is received from themselves and not from their parents. In other words, for participants between 16-18 years of old, the research ethics committee waived the requirement for parental consent. If we understood the meaning of the honorable reviewer correctly, 24 participants were below the age of 18.

3. Study strengths: I would recommend describing them in narratives rather than just putting bullet points. Kindly correct the validated tools-related information there if it was not the case.

- Our thanks to the honorable reviewer for your attention. We reformatted the study strengths section to a text form with more elaborations. This section is followed by the "Study limitations" section. It should be noted that the tools used in this study were translated and validated in Persian by Iranian researchers ("Research tools" section).

4. I was concerned on the exclusion criteria also included people with psychological disorders such as obsessive-compulsive disorder and unwillingness to participate in the study. Please clarify what was the process of identifying these disorders. was it the beneficiary’s self-disclosure or did the researchers assess their disorders before the research participation? The process is not mentioned in the description. However, it would be nice to avoid such situations as disclosing personal mental health status just with researchers without a follow-up on the situation of the respondent is often not a fair treatment to the research participants. It is recommended to clarify this issue, whether there was any debriefing or message with resources on seeking mental health services or not.

- Our thanks to the honorable reviewer for your attention. We assess this criterion by a self-report question at the beginning of the questionnaire. Since we did not have access to the medical records of the participants, we relied on their self-reports (people who suffer from psychological disorders are mostly aware of their illness, and we assumed that these people are honest in answering this question regarding having psychological disorders). Individuals who confirmed that they do not have psychological disorders, their answers to the questions were examined and analyzed. If we understood the meaning of the honorable reviewer correctly regarding the last part of this comment, it should be noted that at the end of the questionnaire, in the "thanksgiving" section, we asked the participants (who were willing to know the result of their test) to enter their contact number in the box. After evaluating their responses, they were informed of their test's results and received a free phone consultation.

In summary, the research is well designed, the data analysis process and findings of the research are well articulated, and the research findings can benefit other researchers/service providers in future. Thanks to the efforts of the authors for their efforts in this research.

- Our thanks to the honorable reviewer for your complimentary comments and suggestions.
